# Models for Studying the Distribution of Ticks and Tick-Borne Diseases in Animals: A Systematic Review and a Meta-Analysis with a Focus on Africa

**DOI:** 10.3390/pathogens10070893

**Published:** 2021-07-14

**Authors:** Olivier M. Zannou, Achille S. Ouedraogo, Abel S. Biguezoton, Emmanuel Abatih, Marco Coral-Almeida, Souaïbou Farougou, Kouassi Patrick Yao, Laetitia Lempereur, Claude Saegerman

**Affiliations:** 1 Research Unit in Epidemiology and Risk Analysis applied to veterinary sciences (UREAR-ULg), Fundamental and Applied Research for Animal and Health (FARAH) Center, Department of Infectious and Parasitic Diseases, Faculty of Veterinary Medicine, University of Liège, 4000 Liège, Belgium; olivier_mahuton@yahoo.fr; 2 Laboratory of Parasitology and Parasitic Diseases, Fundamental and Applied Research for Animal and Health (FARAH) Center, Faculty of Veterinary Medicine, University of Liège, 4000 Liège, Belgium; achillouedraogo@gmail.com (A.S.O.); laetitia.lempereur@health.fgov.be (L.L.); 3 Vector-borne Diseases and Biodiversity Unit (UMaVeB) of International Research and Development Centre on Livestock in Sub-humid Areas (CIRDES), Bobo Dioulasso 01 BP 454, Burkina Faso; babels005@yahoo.fr; 4 Department of Applied Mathematics, Computer Sciences and Statistics, University of Gent, 9000 Gent, Belgium; Emmanuel.Abatih@UGent.be; 5 Grupo de Bio-Quimioinformatica, Universidad de Las Américas, Quito 170122, Ecuador; marco.coral@udla.edu.ec; 6 Communicable Disease Research Unit (URMaT), Polytechnic School of Abomey-Calavi, University of Abomey-Calavi, Cotonou 01 BP 2009, Benin; s.farougou@gmail.com; 7 UFR Biosciences, Université Félix Houphouët-Boigny, Abidjan 22 BP 582, Cote d’Ivoire; ykpatrick@yahoo.fr

**Keywords:** systematic review, meta-analysis, modeling, ticks, tick-borne diseases, Africa

## Abstract

Ticks and tick-borne diseases (TTBD) are constraints to the development of livestock and induce potential human health problems. The worldwide distribution of ticks is not homogenous. Some places are ecologically suitable for ticks but they are not introduced in these areas yet. The absence or low density of hosts is a factor affecting the dissemination of the parasite. To understand the process of introduction and spread of TTBD in different areas, and forecast their presence, scientists developed different models (e.g., predictive models and explicative models). This study aimed to identify models developed by researchers to analyze the TTBD distribution and to assess the performance of these various models with a meta-analysis. A literature search was implemented with PRISMA protocol in two online databases (Scopus and PubMed). The selected articles were classified according to country, type of models and the objective of the modeling. Sensitivity, specificity and accuracy available data of these models were used to evaluate their performance using a meta-analysis. One hundred studies were identified in which seven tick genera were modeled, with *Ixodes* the most frequently modeled. Additionally, 13 genera of tick-borne pathogens were also modeled, with *Borrelia* the most frequently modeled. Twenty-three different models were identified and the most frequently used are the generalized linear model representing 26.67% and the maximum entropy model representing 24.17%. A focus on TTBD modeling in Africa showed that, respectively, genus *Rhipicephalus* and *Theileria parva* were the most modeled. A meta-analysis on the quality of 20 models revealed that maximum entropy, linear discriminant analysis, and the ecological niche factor analysis models had, respectively, the highest sensitivity, specificity, and area under the curve effect size among all the selected models. Modeling TTBD is highly relevant for predicting their distribution and preventing their adverse effect on animal and human health and the economy. Related results of such analyses are useful to build prevention and/or control programs by veterinary and public health authorities.

## 1. Introduction

Ticks are of veterinary, medical and economic importance because of their involvement in the transmission of various pathogens to animals and humans. Ticks carry a wider variety of pathogenic micro-organisms than any other arthropod. They are among the most important vectors of diseases affecting animals and humans [1].

Tick-borne diseases (TBD, e.g., theileriosis, babesiosis, anaplasmosis, cowdriosis, lumping skin disease) and tick-associated diseases (e.g., dermatophilosis) represent the main health and management problems of livestock in many developing countries. For example, in Tanzania, TBD have led to significant economic losses in cattle farming. Kivaria et al. in 2006 estimated the economic losses due to TBD in Tanzania at USD 364 million. This loss included cattle deaths estimated at 1.3 million head of cattle or 7.34% of the herd [2]. 

The most important Ixodidae tick species hampering livestock improvement in West Africa belong to the genera of *Hyalomma* spp., *Rhipicephalus (Boophilus)* spp., *Rhipicephalus* spp. and *Amblyomma* spp. [3]. Among these species, *Rhipicephalus microplus* was the most studied during the last two decades following its introduction in Côte d’Ivoire [4] and Republic of Benin [5] in 2002–2004. The other tick species most studied in West Africa is *Amblyomma variegatum* with its transmitted pathogens, especially *Ehrlichia ruminantium*.

The challenging epidemiology of TBD is closely linked to and dependent on environmental factors that impact host accessibility, vector richness and pathogen acquisition [6].

The rapid spread of ticks and TBD suggests the necessity of implementing prompt and efficient preventive and/or curative programs. Developing relevant control programs for tick spread and the various TBD requires a deep understanding of the epidemiology of these ticks and TBD through accurate epidemiological models.

A model is always a simplification of a complex system to improve understanding. There are several types of models, ranging from simple deterministic mathematical models to complex, spatially explicit stochastic simulation models. The approach and design used may vary depending on the degree of understanding of the epidemiology of a particular disease, the quantity and quality of the data available and the experience of the modelers [7]. Modeling is a very valuable decision-making tool for the development of animal disease management policies. It is currently a widely used tool to enhance the effectiveness of disease surveillance activities and to facilitate their evaluation [8]. The choice of model type depends on the objectives of the modeler or the study. In the field of animal health, models can be used in a variety of ways. Models can be used for retrospective analysis, contingency planning, resource planning, training, surveillance targeting and real-time decision support [9]. Taylor [9] has described six types of models according to the objectives of animal health: risk, analytical, disease, population, economic and specialized models. Some of these types of models have already been used or not used to study ticks and tick-borne diseases in the veterinary field. As ticks are living organisms with particular environmental requirements depending on their species, understanding their global distribution often requires the use of species distribution models [10,11]. These species distribution models can be used to assess the suitability of different habitats for certain species and to make decisions about the control of these ticks and the pathogens they transmit [12,13].

This review aimed to collect, evaluate and synthesize the existing knowledge on modeling of the distribution or the spread of ticks and TBD in animal health during the last 20 years. The meta-analysis was implemented based on the values of sensitivity, specificity and the accuracy of the models to assess their performance.

In the literature, we did not find works that were interested in a systematic review of models used to study the distribution of ticks and tick pathogens. The studies that have conducted systematic reviews of work on ticks and TBD are recent. Generally, they focus on the distribution of single tick species or pathogens [14], on risk factors for TBD, on the economic impact of ticks and their pathogens on livestock [15]. A synthesis of the modeling of tick and pathogen distribution was necessary to highlight the different tools available in the field with an emphasis on their performance, hence the relevance and originality of our study.

## 2. Results

**Figure 1:**
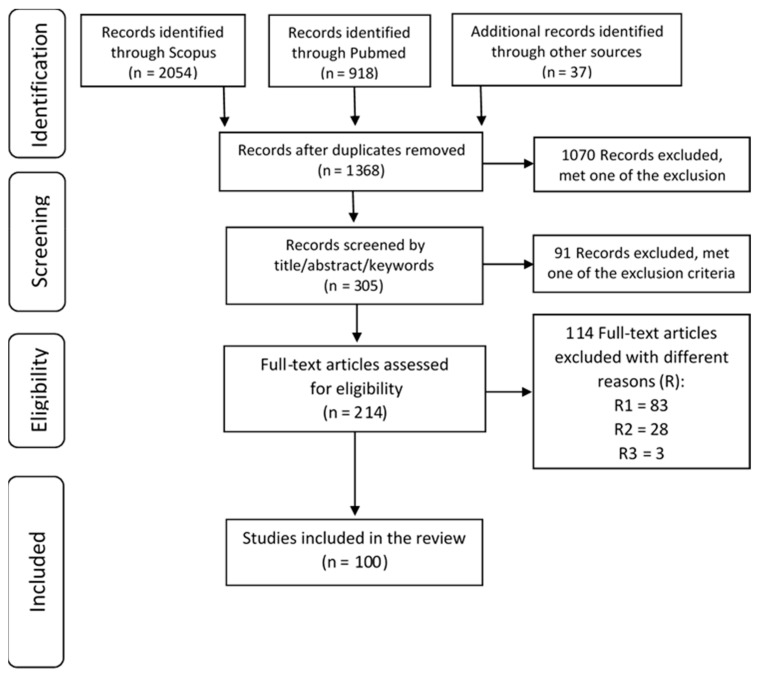
The Preferred Reporting Items for Reviews and Meta-Analysis (PRISMA) Flow Diagram. R1: Records without a clearly stated model; R2: Records with model explanations without field application; R3: Records with a compilation of case studies.

The keywords and Boolean operators used in the request in the two online databases selected 3009 articles, 2054 from PubMed and 918 from Scopus. Other sources (i.e., articles found in the references of the other ones) allowed us to collect an additional 37 articles. The Preferred Reporting Items for Reviews and Meta-Analysis (PRISMA) process in our study helped to, finally, select 100 articles. The PRISMA process results are depicted in Figure 1.

### 2.1. General Description of the Included Studies

**Figure 2:**
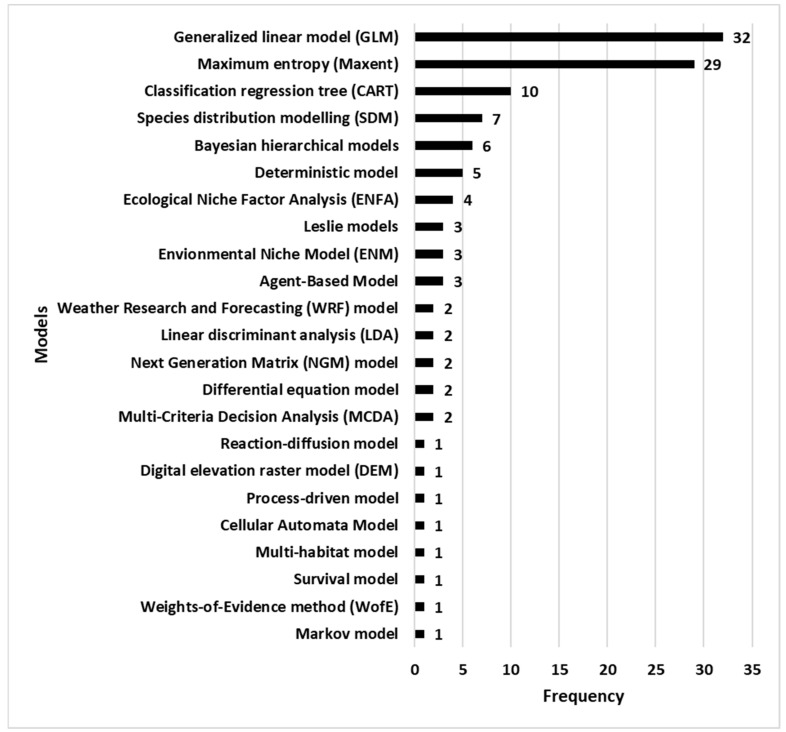
Frequency of models used in the selected publications (decreasing order).

This study selected 100 papers about 23 different models. The most used being the generalized linear model (GLM) with a frequency of 26.67%. The second most frequently used model was the MaxEnt model with a frequency of 24.17% (Table 1 and Figure 2).

The included papers in the analysis of this systematic review were grouped into four different categories: (1) articles modeling tick distribution or spread, (2) articles modeling TBD distribution or spread, (3) models with a sensitivity/specificity analysis and an accuracy analysis, (4) models without any sensitivity/specificity analysis or accuracy calculation. These categories are not mutually exclusive as some papers may model both the distribution of ticks and the pathogens transmitted by these ticks. These papers may analyze performance for some models and not for others. They will therefore be classified in several categories depending on the models.

**Figure 3:**
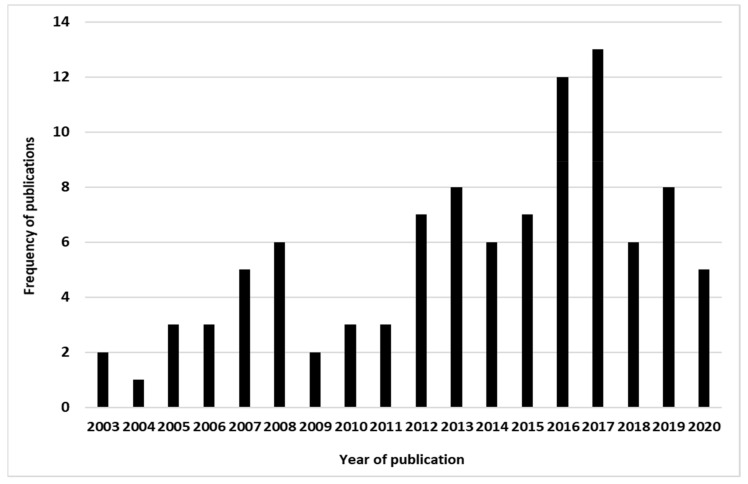
Publication trend on modeling ticks and tick-borne diseases during the last 20 years.

**Figure 4:**
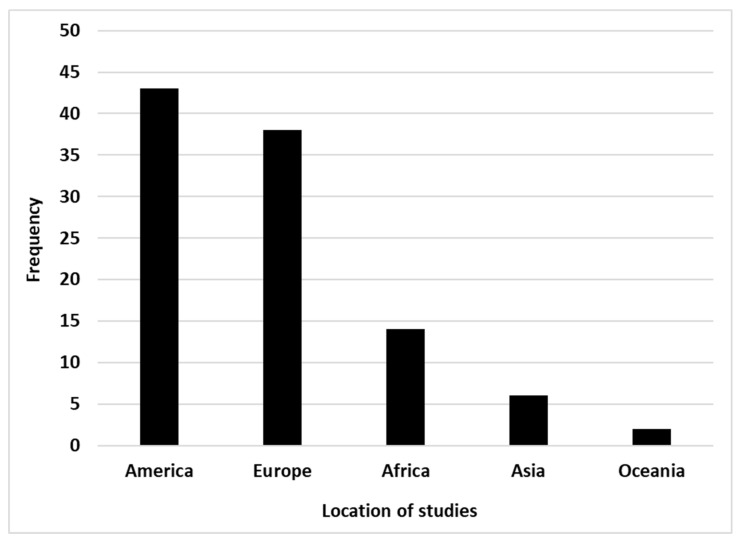
Geographic distribution of the selected publications on ticks and tick-borne diseases modeling.

The number of publications that focused on modeling ticks and TBD increased over time, which could reflect increased scientific attention or interest (Figure 3). America (43%) and Europe (38%) are areas more concerned by this modeling but Africa (14%) has also an interest in this research subject (Figure 4). Asia and Oceania are the less concerned by TTBD modeling with, respectively, 6% and 2% of the selected publications. Considering our study period (2001–2020), the first modeling of ticks and TBD in Africa took place in the first half of our study period, i.e., in 2003. The most recent tick modeling study over our study period was done in 2019. Oceania appeared as a continent less concerned by TTBD modeling. At the country level, the United State of America (USA) was the area the more concerned with modeling ticks and their transmitted pathogens. Globally, the majority of the studies took place in developed countries.

### 2.2. Modeling Ticks Distribution or Habitats

**Figure 5:**
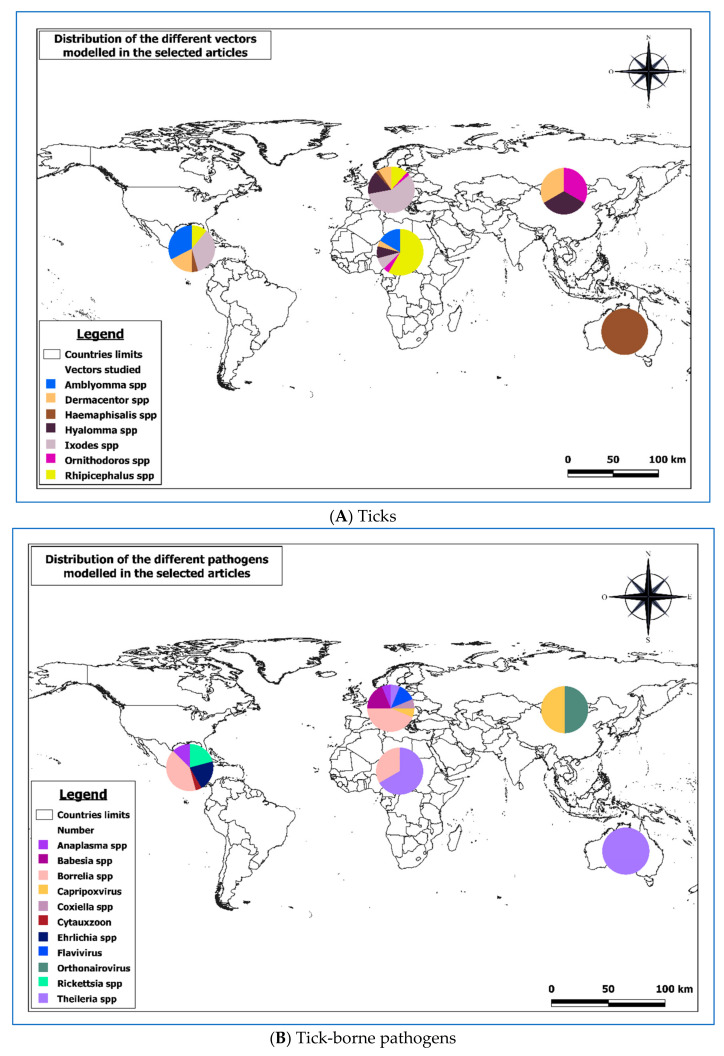
Geographical distribution of the different studies according to the object ((**A**) ticks and (**B**) tick-borne pathogens) of the models.

Seven genera of ticks have been modeled in 86 papers (Table 2 and Figure 5A). The most frequently modeled tick genus is *Ixodes* spp. (41 papers) and modeling areas are essentially in Europe (25 papers) and America (16 papers). The least modeled genus is *Ornithodoros* spp. (1 paper).

The most prevalent tick genus in terms of distribution patterns in Africa was *Rhipicephalus* spp. With 58% followed by *Amblyomma* spp. With 17%. The least prevalent genus in modeling distribution was *Haemaphysalis* spp.

On the American continent, the most prevalent tick genera in modeling were *Ixodes* spp. With 35% followed closely by *Amblyomma* spp. (33%). The least prevalent genera in distribution modeling were *Hyalomma* spp. And *Ornithodoros* spp.

In Asia, only the genera *Dermacentor* spp., *Hyalomma* spp. And *Ornithodoros* spp. Were modeled with a prevalence of 33% each.

In Europe, it was like in America with the genus *Ixodes* spp. (58%) followed by the genus *Hyalomma* spp. With 16%. The least prevalent genus in terms of modeling the distribution in Europe was *Amblyomma* spp.

In Oceania, only the genus *Haemaphysalis* spp. Has been modeled (Figure 5B).

Overall, these results showed that North America and Europe are more adapted to tick species of the genus *Ixodes* spp., notably *I. scapularis* and *I. pacificus* for North America and *I. icinus* for Europe. Africa is much more suitable for tick species of the genus *Rhipicephalus* spp. Ticks of the genus *Amblyomma* spp. do not seem to be widespread in Europe, unlike in America and Africa, where they seemed to be quite present.

### 2.3. Spatio-Temporal Modeling of Tick-Borne Diseases

Eleven genera of pathogens transmitted by ticks have been modeled, representing 39 papers (Table 3 and Figure 5B). The most modeled pathogen is *Borrelia* spp. (18 papers) and the modeling areas are essentially in America (10 papers) and Europe (7 papers). A similar distribution was observed for the genus *Ixodes* spp. This similarity is mainly due to the fact that *Ixodes* tick (*Ixodes ricinus* and *Ixodes scapularis*) is known to transmit *Borrelia* spp. as well. The most modeled pathogen genus in Africa is *Theileria* spp. transmitted essentially by the ticks of the genus *Rhipicephalus* spp.

### 2.4. Models with Estimation of Sensitivity, Specificity or Accuracy Values

**Figure 6:**
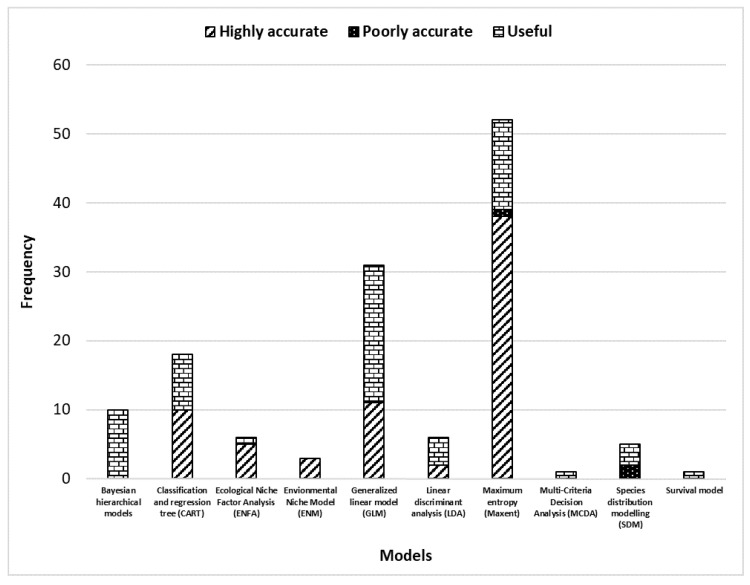
Classification of the models based on the area under the curve (AUC) of a receiver operating characteristic (ROC) plot as the measure of prediction success using the scale established by J.A. Swets in 1988.

Some of the selected publications have evaluated the predictive performance of their models by calculating sensitivity, specificity and/or the accuracy. Models that have a high sensitivity and specificity are supposed to well predict the presence (sensitivity) and the absence (specificity) of either the ticks or the associated pathogens. The study showed 39 papers with the value of the accuracy of the model given by the area under the curve (AUC) of a receiver operating characteristic (ROC) plot as the measure of prediction success, i.e., accuracy (Figure 6; Appendix C). Only nine papers provided values of sensitivity and/or specificity of the model (Table 4).

Most of the models that tested the predictive performance were classified as at least “useful” (AUC ≥ 0.7). Indeed, it is supposed that the models that are not useful are not published or the value of sensitivity is not clearly stated. The most commonly used model is the GLM but the best model according to the robustness analysis is the MaxEnt model. The MaxEnt model was classified on its robustness as (in a decreasing order) highly accurate (in 73.08% of cases), useful (in 25% of cases), and poorly accurate (in 1.92% of cases). While the GLM model was classified on its prediction accuracy as (in a decreasing order) useful (in 64.52% of cases) and highly accurate (in 35.48% of cases) (Figure 6). The papers that use the MaxEnt model stated the accuracy at 75.86% while those that use the GLM stated the accuracy at 37.5%.

### 2.5. Models without Any Estimation of Sensitivity, Specificity and Accuracy Values

The majority of the selected models included in 48 different papers did not mention any sensitivity, specificity or accuracy calculation to show the performance of their models or the authors do not clearly state this analysis (Table 5).

### 2.6. Focus on Modeling Ticks and Tick-Borne Pathogens in Africa

**Figure 7:**
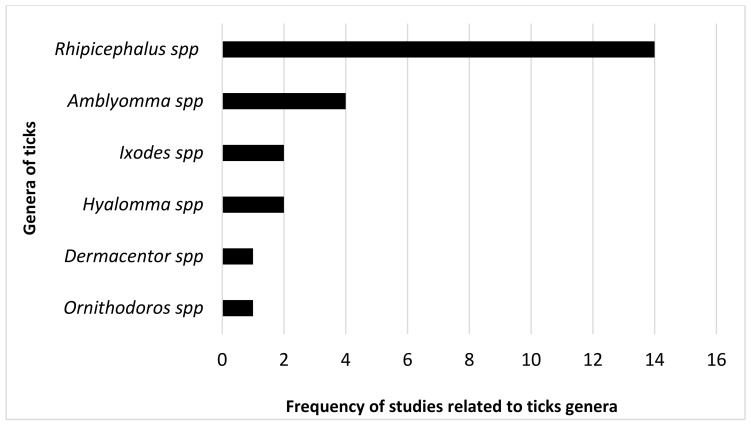
Genera of ticks modeled in Africa based on this systematic review.

Six genera of ticks (*Amblyomma* spp., *Dermacentor* spp., *Hyalomma* spp., *Ixodes* spp., *Ornithodoros* spp. and *Rhipicephalus* spp.) and two genera of pathogen transmitted by ticks (*Borrelia* spp. and *Theileria* spp.) have been modeled in Africa (Figure 7 and Table 6). The most modeled tick species in selected studies in Africa is *Rhipicephalus appendiculatus* also called “Brown ear tick” or “African tick”. The invasive tick *Rhipicephalus (Boophilus) microplus*, has been modeled in Tanzania in Eastern Africa (2008), in Republic of Benin in Western Africa (2013 and 2015), in a more general study in Africa (2009) and recently in Zimbabwe (2018). The models that were used are presented in Table 6.

### 2.7. Meta-Analysis of the Models’ Accuracy

**Figure 8:**
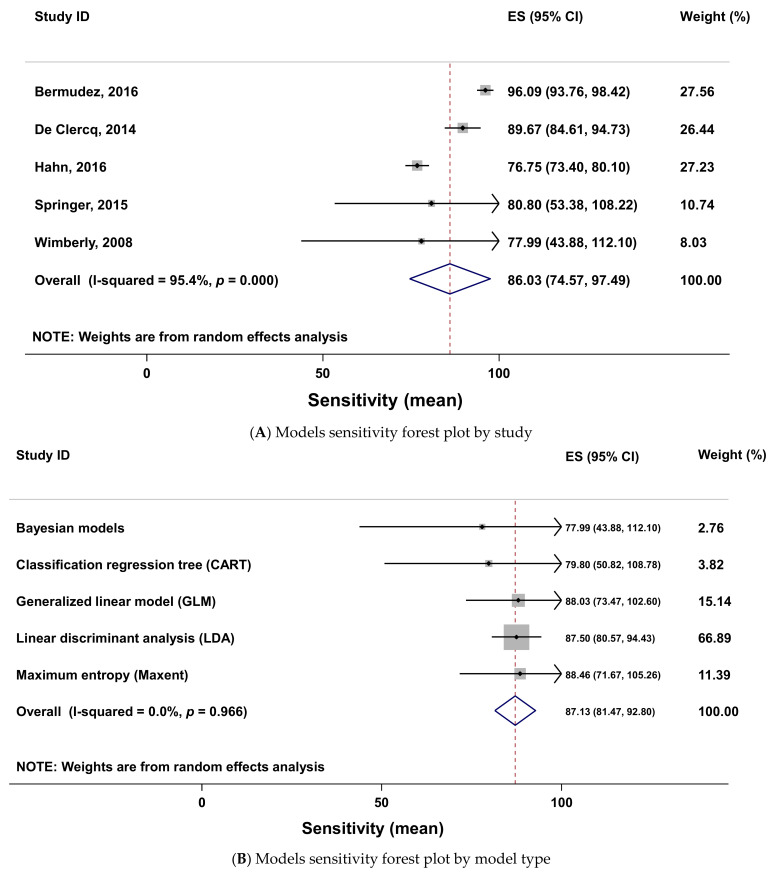

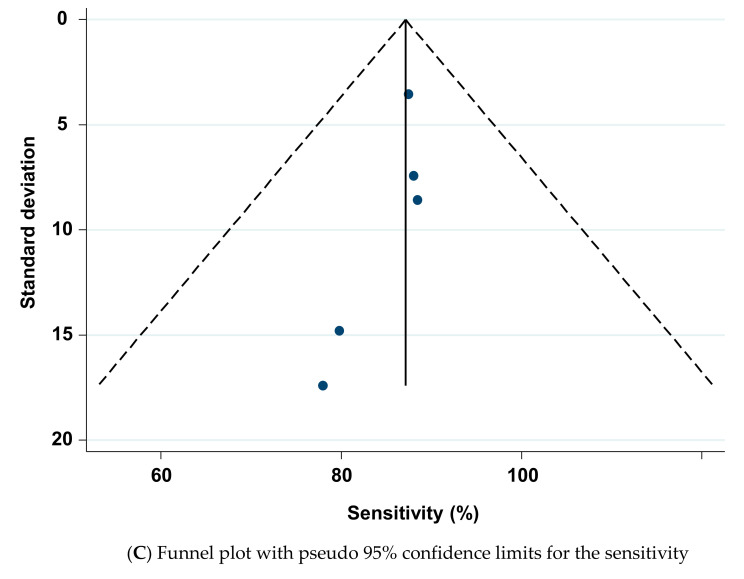
Forest plots (**A**,**B**) and funnel plot (**C**) of the sensitivity of models using meta-analysis. Forest plot legend: ES (effect size); CI (confidence interval); names on the left (first author of primary studies); solid line (sensitivity mean); grey square size (weight of each study); horizontal lines (95 confidence intervals); vertical line (line of no effect); diamond (overall sensitivity effect); vertical dash line (combined sensitivity effect); tips of diamond (95% confidence intervals).

This meta-analysis was possible with 20 of the 100 worldwide selected articles. The meta-analysis with sensitivity data was done with five studies. The forest plot shows that the sensitivities have different effect sizes in the various studies (Figure 8a). All effect sizes (ES) were positive and significant because none of their confidence intervals include zero. The study with the highest weight in this meta-analysis is that of Bermudez [16]. The one with the lowest weight is the study of Wimberly [102]. This meta-analysis revealed a very high degree of heterogeneity in the sensitivities ES (heterogeneity chi-squared = 87.20 (d.f. = 4) *p* < 0.001; I-squared (variation in ES attributable to heterogeneity) = 95.4%; test of ES = 0, z = 14.71, *p* < 0.001). This information suggests that there are some subgroups of sensitivities (i.e., type of models) in these studies. A meta-analysis by type of models was needed to check it. The meta-analysis considering the type of models as subpopulations showed a homogeneity (heterogeneity chi-squared = 0.57 (d.f. = 4) *p* = 0.966; I-squared (variation in ES attributable to heterogeneity) = 0.0%; test of ES = 0, z = 30.13, *p* < 0.001) with the sensitivity data (Figure 8b). The funnel plot (Figure 8c) confirmed this homogeneity. This funnel plot showed a symmetrical shape that means an absence of publication bias in the studies. It also showed that among the five models studied in this meta-analysis there was one that was very precise with a small standard deviation and located at the top of the funnel on the graph. Three models are very close to the average, proof of their very good precision (Figure 8c). There are three models (MaxEnt, GLM and LDA) in which sensitivity effect sizes are greater than the pooled effect size of the meta-analysis (Figure 8b). The MaxEnt model has the highest sensitivity effect size (i.e., 88.46).

**Figure 9:**
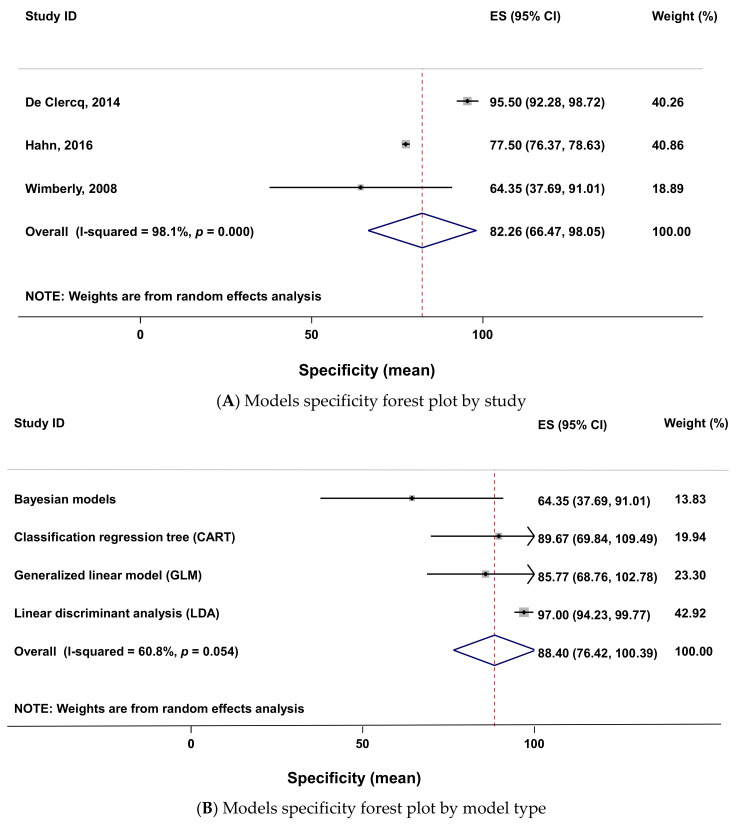
Forest plots (**A**,**B**) of the specificity of models using meta-analysis. Forest plot legend: ES (effect Size); CI (confidence interval); names on the left (first author of primary studies); solid dot (specificity mean); grey square size (weight of each study); horizontal lines (95 confidence intervals); vertical line (line of no effect); diamond (overall specificity effect); vertical dash line (combined specificity effect); tips of diamond (95% confidence intervals).

The meta-analysis of specificity data was done with three studies. The forest plot of this meta-analysis (Figure 9A) also showed a high heterogeneity (heterogeneity chi-squared = 108.05 (d.f. = 2), *p* < 0.001; I-squared (variation in ES attributable to heterogeneity) = 98.1%; test of ES = 0, z = 10.21, *p* < 0.001). The analysis done considering the type of model as subgroups and plotted on Figure 9B showed a moderate heterogeneity in the specificity effect size (heterogeneity chi-squared = 7.66 (d.f. = 3), *p* = 0.054; I-squared (variation in ES attributable to heterogeneity) = 0.0%, test of ES = 0, z = 14.46, *p* < 0.001). Among the four model groups, the linear discriminant analysis (LDA) and the CART models specificity effect size are greater than the pooled one (Figure 9B). The LDA had the greatest specificity effect size (i.e., 97) and the Bayesian models had the lowest specificity (i.e., 64.35).

**Figure 10:**
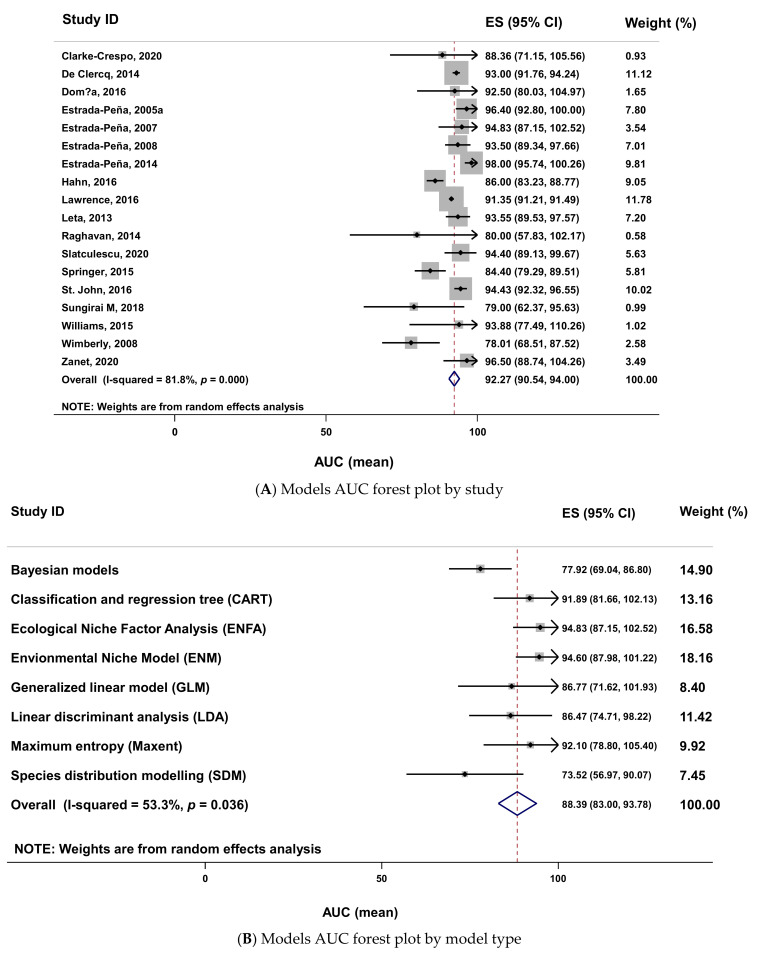
Forest plot (**A**,**B**) of the AUC of models using meta-analysis. Forest plot legend: ES (effect size); CI (confidence interval); AUC (area under the curve); names on the left (first author of primary studies or model’s name); solid dot (AUC mean); grey square size (weight of each study); horizontal lines (95 confidence intervals); vertical line (line of no effect); diamond (overall AUC effect); vertical dash line (combined AUC effect); tips of diamond (95% confidence intervals).

The AUC data meta-analysis was possible with 18 studies. There is a high heterogeneity (Figure 10A) in the effect size of the AUC data (heterogeneity chi-squared = 93.42 (d.f. = 17), *p* < 0.001; I-squared (variation in ES attributable to heterogeneity) = 81.8%; test of ES = 0, z = 104.62, *p* < 0.001). The analysis with the groups of models revealed a moderate heterogeneity (heterogeneity chi-squared = 14.98 (d.f. = 7), *p* = 0.036; I-squared (variation in ES attributable to heterogeneity) = 53.3%; test of ES = 0, z = 32.13, *p* < 0.001). There are four models (ENFA, ENM, MaxEnt and CART) in which effect sizes are greater than the pooled one (Figure 10B). The model with the highest AUC effect size was the ecological niche factor analysis (ENFA).

The large heterogeneity shown in the meta-analysis results of the sensitivity, the specificity and the AUC of the models at the study level shows the large uncertainty in the spatial distribution of ticks and TBD. This uncertainty was reduced considerably when the meta-analysis is done according to the types of models. The analysis is, therefore, better when done according to homogeneous groups of models.

## 3. Discussion

This study is the first systematic review of tick and tick-borne disease distribution models in animals with an assessment of the performance of these models through a meta-analysis. This study is also original in that it has a small focus on Africa following a global review of tick and tick-borne disease distribution modeling worldwide.

### 3.1. Models Used in the Selected Papers

The three models mostly used are the generalized linear model (GLM) with a frequency of 26.67%, the MaxEnt model with a frequency of 24.17% and the classification and regression tree (CART) with a frequency of 8.33%.

The GLM is a mathematical extension of the linear model that does not force data into unnatural scales and thereby allows for non-linearity and non-constant variance structures in the data. The data can be assumed to come from several groups of probability distributions, such as normal, binomial, Poisson, negative binomial or gamma, many of which better match the non-normal error patterns of many environmental data. This makes the GLM more accommodating and adaptable to the analysis of ecological linkages, that can be misrepresented by conventional Gaussian probability distributions [117]. In many standard statistical techniques, the absence/presence occurrence data are required for predictive modeling of species environmental requirements and geographic distributions. The use of spatial variables such as geographic coordinates is evident in machine learning models such as MaxEnt. However, these spatial data should be given special attention when used in regression models such as GLMs in species distribution modeling. The selection of predictors should also be given special attention to avoid collinearity, which should be seriously investigated. Predictors that show strong collinearities should be removed from the final model. Several processes exist for the analysis of collinearities. One of them is the variance inflation factor (VIF).

The MaxEnt method is a recent species geographic distribution modeling approach, which requires only presence data. High predictive accuracy is achieved with this model in addition to some interesting characteristics. The performance of the MaxEnt model is influenced by a limited set of factors [118]. It is a method of machine learning for general use. MaxEnt uses a straightforward and precise mathematical approach with certain aspects that make it well adapted to the modeling of species distribution [119]. A tick species absence in a geographic area is difficult to assert and needs very long and important investigations. The use of the MaxEnt model does not need great computer skills. That is certainly why many authors prefer to use this model. The MaxEnt model has a native probabilistic meaning, providing a gentle gradient from the most suitable to the least suitable conditions. The model may be conveniently explained by specialists, a characteristic of high functional relevance [120]. Some weaknesses have been identified with this model. These weaknesses concern the model transferability, the model evaluation and its selection. With the MaxEnt model, it is difficult to transfer results from one sampled area to another non-sampled area. This transferability could be a major problem since environmental variables vary by species. It is therefore important to do a good sampling in time and space (i.e., invasive species such as *Boophilus microplus*) to build good predictive models. It is therefore important to be careful about sampling bias [121]. When a model is built, this model is not necessarily informative. One of the biggest challenges MaxEnt faces is related to the evaluation of the model and the subsequent choice of model. Various approaches (i.e., AUC, Kappa, and Jackknife statistics) have been used to assess the importance of the models developed, although it is not clear which one is the most appropriate and whether they can help in the choice of the model [121]. The process of evaluation of models is very useful to know when a model can well predict the distribution of species. On the other hand, the evaluation process is not very useful to select the best model. The selection process is often based on one of these two approaches: the AUC approach and the Jackknife test. The AUC approach used by Hoenes and Baldwin [122,123] is based on the AUC score of listed models built from the most general to the parsimonious one. The best model selected is the one with the least variables with the best AUC score. The other selection approach is the Jackknife test of variable importance. This test is used to appreciate the strengths of each predictor variable of the model [124]. The principle of the Jackknife test is to remove one by one the predictor variable and appreciate the decrease in training gain when omitted. If the omission of a variable did not have a significant decrease on the average training gain it is removed from the model.

The third most frequently used model, the CART (classification and regression tree) model, also has strengths and weaknesses. The CART model is suitable for exploring data sets and can readily recognize linkages between factors. In contrast to logistic regression methods, CART has no requirement to specify the function used to analyze the covariates. These advantages are particularly valuable in dealing with the data non-uniformity (data from different sources) commonly associated with field data sets. The CART model is, however, vulnerable to incomplete data, which can be a problem when dealing with projectively sampled datasets [125]. There are some algorithms (the K-nearest neighbor algorithm, the E.M algorithm, the C4.5 algorithm and the CN2 algorithm) that can deal with the influence of missing data on the accuracy of a machine learning prediction [126].

Two types of models, depending on the variability and the uncertainty of data, are distinguished. Models that assign the mean, or the most probable value to all factors and models, which assign the mean or the most expected outcome of likely events, are called, deterministic models. They generate a unique outcome or response for each group of entry values [9]. Deterministic models are used in the following papers (e.g., [127,128]). Models that include variation and the effect of randomness in the approach are referred to as stochastic. Since the values of the model parameters are subject to variation and the arrival of probable events is random, stochastic models should be executed in a serial manner and generate a set of results from the same input case [9]. Stochastic models are used in the following papers (e.g., [119,129]). In addition, the treatment of time and space in the model can also lead to the definition of its type. The choice of a model depends on many parameters such as the object of modeling, the area where the model is supposed to be implemented, the data available, and the resources dedicated to the collection of data. All these parameters should be taken into account in the choice of the optimal and adapted model.

### 3.2. Performance of Models

The most accurate model was the MaxEnt model, which can accurately predict species environmental requirements and geographic distribution [119]. The most frequently used model, the generalized linear models (GLM) seems to be less suitable for modeling species distribution compared to the MaxEnt model. Other models such as classification and regression tree (CART), ecological niche factor analysis (ENFA) and environmental niche model (ENM), developed in some publications, also have at least an acceptable level of accuracy. Information on the performance of models is not always available in all studies on modeling the distribution of ticks and TBDs. The readers need to know about the quality of models developed in various studies. The authors should make this information available in their papers. Information on the performance parameters of the models is crucial in choosing the right model and assessing the quality of the message they provide. This information is essential to give relevance to the decisions of authorities in charge of managing ticks and TBD problems.

### 3.3. Evolution in Time of the Number of the Ticks and Tick-Borne Disease Modeling Studies

This systematic review highlights the increasing interest of scientists in the modeling of species environmental requirements and their geographic distribution (Figure 3 and Figure 4). It has also confirmed the continuous interest of governments, farmers and other stakeholders who provide funding for the development of such models. Ticks and TBD have both medical and economic importance, and this can be noticed through the huge economic losses caused to breeders and the various diseases that can transmit from animals to humans. A better understanding of these environmental requirements and geographic distribution can lead to better management of their control and/or eradication. Climate change as a driver also favors changes in the behavior of these parasites (ectoparasites and hemoparasites). Therefore, the various models help to know the adaptability of ticks to climate changes and forecast their distribution according to this adaptability to further changes.

### 3.4. Geographical Distribution of the Ticks and Tick-Borne Disease Modeling Studies

The large majority of the studies were conducted in developed countries (America and Europe). These results highlight the fact that ticks and TBD get more attention from scientists and funders in developed countries than developing ones. Currently, international organizations sponsor most research programs in developing countries and many projects are implemented using different north–south partnerships. However, the abundance and burden of ticks and TBD are more important in developing countries and the subsistence of the local population is more dependent on its livestock [130]. Moreover, transhumance is a traditional practice, which has an impact on the spread of ticks and TBD [131,132]. Due to these reasons, the governments of developing countries should pay more attention to the funding of scientific national or regional institutions to conduct more research on ticks and TBD modeling as a decision tool for management purposes. In addition, associated socio-anthropology studies are needed to understand the perception of local populations concerning ticks and TBD.

Most studies do not mention the exact geographical location of the collection points of the analyzed samples on which they have based their models [85,116]. In the majority of cases, the localities (city, country, and region) were mentioned, without specifying the geographical coordinates of the collection points [53,101,105,112]. This situation has made it difficult to map the distribution of the various species studied. The availability of these data would have made it possible to carry out a meta-analysis much more oriented towards the presence and absence of ticks and tick-borne diseases than towards the performance of the models.

### 3.5. Ticks and Pathogens Modeled

*Ixodes* spp. (*Ixodes scapularis*, *Ixodes pacificus* and *Ixodes ricinus*) and *Borrelia* spp. (*Borrelia burgdorferi* sensu lato) are, respectively, the ticks and the pathogens most frequently modeled in the selected articles. *Ixodes scapularis* and *Ixodes pacificus* are the primary vectors of *Borrelia burgdorferi* s.l. responsible for Lyme disease, one of the most prevalent TBD in the United States [71]. The tick *Ixodes ricinus* is responsible of the transmission of the pathogens that cause tick-borne encephalitis (TBE) and Lyme disease [133,134,135] in northern Europe. This review also showed that more than 75% of the models have been developed in this area (America and Europe). This situation is logical because the majority of the studies are located in northern (developed) countries. In these countries, the main species of ticks that has economic and medical importance is the *Ixodes* spp. and their transmitted pathogens.

According to our study, the most modeled tick genus in Africa is *Rhipicephalus* spp. (60%). Overall two genera of pathogens (*Theileria* spp. (75%) and *Borrelia* spp. (25%)) transmitted by the ticks have been modeled by our selected articles. *Rhipicephalus appendiculatus* is known as the African tick and is the vector of *Theileria parva* in eastern, central and southern Africa [136,137]. After the first detection of the invasive tick, *Rhipicephalus* (*Boophilus*) *microplus* in the southern part of Cote d’Ivoire [4] and Benin [5] it has spread into these two countries and to other neighboring West African countries like Burkina Faso, Mali, Niger, Nigeria and Togo [138]. All these facts highlight that this genus of tick (*Rhipicephalus* spp.) has a huge economic and medical importance in Africa and thus deserves serious attention for its control and/or eradication.

The less frequently modeled genus according to this study is the genus *Ornithodoros* spp. This genus hosts the species of soft ticks involved in the transmission of African Swine Fever (ASF). ASF has resurfaced and is spreading nowadays in Asia and Europe and is becoming a major concern for domestic and wild swine in these countries. This disease is still endemic in many West African countries and the role of the *Ornithodoros* spp. tick species in the persistence of ASF is not yet clearly established. More investigations are needed to clarify the role of *Ornithodoros* spp. in the persistence, the resurgence and the spread of ASF.

Lumpy skin disease (LSD) is an emergent and financially damaging viral illness of livestock. The infection is presently endemic in the majority of African countries and has been spreading beyond Africa to the Middle East region lately [139]. The principal means of transmission of the virus responsible for this disease is believed to be by hematophagous arthropod vectors such as ticks. This review did not get find article satisfying our criteria and that models the lumping skin disease. Therefore, these diseases (i.e., AFS and LSD) should be given more attention in the future.

### 3.6. Focus on Modeling Ticks and Tick-Borne Diseases in Africa

Six genera of ticks have been modeled in Africa among which the genus *Rhipicephalus* spp. is the most frequent. Within this genus, two important species are of special interest: *Rhipicephalus appendiculatus* known as the African tick and *Rhipicephalus* (*Boophilus*) *microplus*, a new invasive species resistant to usual acaricides available to breeders. *Rhipicephalus appendiculatus*, also known as the “brown ear tick” because of its color and strong tendency to feed on the ears of cattle, is the principal vector of *Theileria parva*. *Theileria parva* is the parasite that causes East Coast Fever (ECF) in the eastern, central and southern regions of Africa. It is the focus of several studies because of its involvement in East Coast Fever in cattle [140]. This tick is called the “African tick” because of its distribution located exclusively in the Africa continent. The tick *Rhipicephalus appendiculatus* is often found in savannah and temperate climates, ranging from hot coastal areas to cool highlands with a humid climate. This tick is widespread from southern Sudan to the southeast coast of South Africa [136,137,140]. *Theileria parva* is the only pathogen modeled in Africa in the selected articles. This pathogen is transmitted by the African tick *Rhipicephalus appendiculatus*. This pathogen is the cause of East Coast Fever one of the most important TBD in Africa [141]. Many other TBDs namely anaplasmosis, babesiosis and cowdriosis are also constraining to domestic ruminant production in Africa [2,142] and then need attention to understand the process of their spread.

*Rhipicephalus* (*Boophilus*) *microplus* is the recently introduced Asian cattle tick in Africa. It is an invasive tick characterized by a high level of resistance to usual acaricides. As this tick is responsible for the transmission of both *B. bovis* and *B. bigemina*, it represents a potentially higher burden on livestock husbandry than *Rhipicephalus (Boophilus) decoloratus*. *Anaplasma marginale* and *Borrelia theileri* are also transmitted by *Rhipicephalus* (*Boophilus*) *microplus*. *Rhipicephalus* (*Boophilus*) *microplus* needs more attention in the modeling of its distribution in Africa to predict. Since its introduction in West Africa through cattle trade in 2000 in Ivory Coast [4] and 2004 in Benin [5], this tick species had spread notably in many other West African countries [138]. Scientists should pay more attention to the mechanism of the spread of this species through models and suggest its control and/or eradication strategies.

### 3.7. Meta-Analysis of the Models’ Accuracy

The meta-analysis of the sensitivity, specificity and AUC of the various studies that provide these data reveal a very high heterogeneity between the sensitivities, specificities and the AUC of the studies. There are different effect sizes in different types of subgroups. These studies discussed five different types of models concerning the sensitivity data, four types of models concerning the specificity and eight different types of models concerning the AUC data. This variety of model types in the studies leads to the high heterogeneity in the effect size of the sensitivity, specificity and AUC. Therefore, when the meta-analysis was done after grouping the data according to the type of model there was a homogeneity with sensitivity and specificity effect sizes and a moderate heterogeneity with the AUC effect sizes. This means the different categories of models will have similar sensitivity, specificity and AUC effect size in ticks and tick-borne diseases modeling. These results also highlighted the good performance of some models to predict the presence of tick species (MaxEnt, GLM and LDA) according to their sensitivity above the level of the pooled effect size. Results also showed that some models had a better ability to predict the absence of vectors and pathogens species (LDA, CART and GLM) according to their specificity above the pooled effect size. These two models need species presence and absence data to predict their presence in an area. The input of these various models (only presence data for MaxEnt and presence and absence data for CART and GLM) could explain the quality (sensitivity and specificity) of their outputs (prediction).

Overall, the models in the selected publications for meta-analysis had good sensitivity, specificity and accuracy (AUC). Models with poor sensitivity, specificity and accuracy are not published and could lead to an overestimation of the quality of the different types of model. Many studies did not assess the sensitivity, specificity and/or accuracy data. These parameters make it possible to assess the quality of models developed in those studies. Data about the presence or absence of species and details of calculation of sensitivity, specificity and accuracy are often missing in many studies and complicated the meta-analysis of the quality of such models. We recommend that such studies should make available all data related to the building of models to make any meta-analysis of these data feasible.

Models that do not present data to assess their performance are not very useful for tick and TBD control. The performance parameters of the models (sensitivity, specificity and AUC) besides informing about the quality of the models presented also give an idea about the level of risk of the object (tick or TBD) modeled in the area studied. This helps to raise the awareness of the authorities about the need to take urgent action to counter the potential hazard. These parameters can also be used to measure the extent of a TBD or tick species already established in an area. This information is crucial in forecasting the budget and logistics to be mobilized for control or prevention.

## 4. Materials and Methods

### 4.1. Search Strategy and Study Selection

The Preferred Reporting Items for Systematic Reviews and Meta-Analyses (PRISMA) guidelines were followed in this systematic review [118,119]. PRISMA is a minimum set of elements for evidence-based reporting in systematic reviews and meta-analyses. The PRISMA guidelines aim to provide writers with standards for formatting systematic reviews and meta-analyses. The PRISMA guidelines consist of a checklist of 27 items and a four-phase flow chart (Figure 1; Appendix A and Appendix B).

Published articles related to the dynamics of ticks and TBD modeling in animals were downloaded via two online databases: Scopus (www.scopus.com, accessed on 15 January 2021) and PubMed (www.ncbi.nlm.nih.gov/pubmed, accessed on 15 January 2021). The keywords and Boolean operators used were the following: ((modelling or modeling or models) and (animals and ticks) and (tick and borne and diseases) and (distribution or spread) and dynamic)). The references of the retrieved articles and journals were also consulted to see if there were any eligible articles for our study. When there were several articles for the same study, we retained the latest version, supplemented if needed, with information from the most complete version.

### 4.2. Eligibility Criteria

Potentially eligible articles for this systematic review were original articles, written in English or French, published in the period 2001–2020 and that describe models of the distribution of TTBD. Articles that describe biological or economic models instead of spatial and/or temporal distribution models of tick and/or TBD were not included. Details of the inclusion and exclusion criteria are summarized in Table 7.

### 4.3. Meta-Analysis of the Accuracy of Models in the Selected Studies

The selected publications were screened according to an analysis of the performance of their models. This analysis was based on the values of the sensitivity, specificity or accuracy available of some of the models elaborated in these studies. The sensitivity was defined as the proportion of established counties classified as tick-present locations by the model [33]. Sensitivity was also described as the percentage of areas with a known adequate habitat that were ranked as appropriate by the model. Specificity is the percentage of areas where no established tick population was recorded and which were ranked as such in the model [71]. Quantitative measurement was carried out by checking predictions against observations in an unrelated test data set with the area under the curve (AUC) of a receiver operating characteristic (ROC) plot as a gauge of prediction accuracy [143]. According to J.A. Swets [144] cited by Antoine Guisan [145], the accuracy of a model was estimated with the value of AUC. If the AUC value > 0.9, the model is considered as “highly accurate”. Models providing values in the range 0.7–0.9 were considered as “useful”, and those lower than 0.7 “poorly accurate”.

A subsequent meta-analysis was performed with these data (sensitivity, specificity and AUC) to appreciate the quality of the different models developed in the selected articles. A random-effect model was used to see the level of heterogeneity of the means of the sensitivity, specificity and AUC of the selected studies in which these data are available. We used Stata’s “metan” meta-analysis command. This command allows the user to enter the frequencies of the 2 × 2 table cells for each study (for binary results), the mean and standard deviation in each group (for numerical results), or the effect estimate and standard error of each study [146]. In our study, we used the mean and standard deviation of the sensitivity, specificity and AUC data of the models developed in the selected papers. We chose the random effect option because of the diversity of the studies in their methodologies. The heterogeneity in the meta-analysis was quantified by the I2 that is the percentage of variation attributable to heterogeneity [147,148]. According to the classification of Higgins [148] adjectives of low, moderate and high are assigned to I2 values of 25%, 50% and 75%. In the case of high heterogeneity, the existence of subgroups was checked [149,150]. When the homogeneity was obtained, a funnel plot assessed the risk of publication bias. This meta-analysis was performed with Stata 14^®^.

## 5. Conclusions

Modeling ticks and TBD distribution is important to help all stakeholders in animal health and public health to identify high-risk areas of these parasites for animals and humans. Modeling the distribution of these parasites is very useful for authorities to inform decisions for the prevention and/or control programs. In addition, this review highlighted also the importance of vector surveillance and prevention/control in countries that have not yet detected invasive tick species (e.g., *Rhipicephalus microplus*) but are in the areas predicted to host suitable habitats. Indeed, awareness raising and training of different stakeholders must be reinforced for better prevention and control of this tick in these different countries according to their status.

The GLM models appear to be the most used in modeling the distribution of ticks and TBD. The maximum entropy model (MaxEnt) revealed to have good performance in the prediction of the presence of tick species. The particularity of this modeling system is the use of the tick presence data only without the need for the absence data. This aspect is beneficial in tick distribution modeling in Africa where tick absence data are rarely available. Indeed, this kind of model combined with the GLM will be very powerful in ticks and TBD modeling in Africa. Above all, the choice of a model depends on several parameters and criteria that deserve special attention to achieve objectives set during the modeling process.

## Figures and Tables

**Table 1:** Categories and objectives of main modeling techniques collected in this review.

Category	Model	Objective
Regression models	Generalized linear models (GLM)	Investigate the levels of tick aggregation at different spatial ranges, determine and disaggregate the drivers of tick density and probability of presence, and provide robust estimates of tick densities between landscape segments. Study the effect of environmental conditions on the prevalence of different stages of ticks and in the epidemiology of the tick-borne disease (TBD).
Species distribution modeling	Maximum entropy (MaxEnt)	Explore the limits of the potential distribution by extrapolating the environmental requirements of ticks. Analyze the possible spatial range of tick species, to explore how climate changes can shape the distribution of these species.
	Classification and regression tree (CART)	Review data on tick distribution and prevalence of TBD for a national TBD management approach using the current ecological and epidemiological information on ticks and the related diseases they transmit.
	Species distribution modeling (SDM)	Discuss and illustrate the precise boundaries of the present range of ticks based on computational map modeling and demonstrate the way in which local populations of these ticks differ in abundance towards the boundaries of the range.
	Ecological niche factor analysis (ENFA)	Measuring the extent to which the requirements of a given species deviate from average conditions and the extent to which the species is selective over the range of environmental conditions available in a country. Develop a rigorous definition of the climatic niche of a set of relevant tick species in a geographical area.

**Table 2:** Ticks modeled by the selected publications.

Ticks Studied	Country or Continent of Interest	References
*Amblyomma* spp.	Africa, Brazil, Central and Southern America and USA.	[16,17,18,19,20,21,22,23,24,25,26,27,28,29,30,31,32,33,34]
*Dermacentor* spp.	Europe, Czech Republic, Mediterranean region, Panama and USA.	[16,17,34,35,36,37,38,39,40,41,42,43,44]
*Haemaphysalis* spp.	Europe, New Zealand and USA.	[38,44,45,46,47]
*Hyalomma* spp.	Europe, Mediterranean region, Romania, South Africa, Spain and Western Palearctic.	[18,39,44,48,49,50,51,52]
*Ixodes* spp.	Belgium, Canada, Europe, France, Germany, Iceland, Ireland, Italy, Netherlands, Norway, Panama, Scotland, Slovakia, Spain, UK, USA and Western Palearctic.	[17,22,36,38,44,51,53,54,55,56,57,58,59,60,61,62,63,64,65,66,67,68,69,70,71,72,73,74,75,76,77,78,79,80,81,82,83,84,85,86,87,88]
*Ornithodoros* spp.	Africa, Asia and Europe.	[12]
*Rhipicephalus* spp.	Africa, America, Republic of Benin, Djibouti, Eritrea, Ethiopia, Europe, France, Mediterranean region, Panama, Romania, Somalia, South Africa, Tanzania, USA, Zimbabwe.	[16,17,18,22,26,27,36,39,44,50,89,90,91,92,93,94,95,96,97,98,99,100]

**Table 3:** Pathogens modeled according to this systematic review (alphabetic order).

Pathogen	Country or Continent of Interest	References
*Anaplasma* spp.	USA	[62,101,102]
*Babesia* spp.	Europe	[72,84,103]
*Borrelia* spp.	Canada, Italy, Scotland, Slovakia, Spain, USA and Western Palearctic	[55,56,60,62,65,73,80,81,83,85,88,104,105]
*Capripoxvirus*	Asia and Europe	[106]
*Coxiella* spp.	Spain	[107]
*Cytauxzoon* spp.	USA	[108]
*Ehrlichia* spp.	Australia and USA	[24,32,102,105,109]
*Flavivirus*	Europe, Italy and Scotland	[57,110]
*Orthonairovirus*	Iran, Turkey	[111]
*Rickettsia* spp.	Brazil, Panama and USA	[16,22,29,35,43]
*Theileria* spp.	Africa, Djibouti, Eritrea, Ethiopia, Greece, New Zealand and Somalia	[45,46,95,97,103]

**Table 4:** Models with only sensitivity and/or specificity values.

Type of Model	References	Sensitivity (%)	Specificity (%)	Youden Index
Classification and regression tree (CART)	[71]	75	78	0.53
Classification and regression tree (CART)	[91]	89	96	0.86
Classification and regression tree (CART)	[33]	87	-	ND
Generalized linear model (GLM)	[53]	100	80	0.80
Generalized linear model (GLM)	[59]	91.3	96.4	0.87
Generalized linear model (GLM)	[63]	89	82	0.71
Generalized linear model (GLM)	[91]	90	95	0.85
Generalized linear model (GLM)	[33]	88.5	-	ND
Linear discriminant analysis (LDA)	[91]	85	98	0.84
Maximum entropy (MaxEnt)	[16]	95.76	76	0.72
Maximum entropy (MaxEnt)	[71]	79	78	0.57
Maximum entropy (MaxEnt)	[64]	79.5	-	ND
Maximum entropy (MaxEnt)	[33]	84	-	ND
Bayesian hierarchical models	[102]	>90	<60	ND

The Youden index represents the sum of the sensitivity and the specificity (expressed between 0 and 1) minus 1; ND: not determined.

**Table 5:** Models without any sensitivity, specificity or accuracy analysis and their related papers.

Model	Reference
Agent-based model	[23,73,85]
Bayesian hierarchical models	[32,101,105,106,112]
Cellular automata model	[66]
Classification and regression tree (CART)	[26,87,96]
Deterministic model	[48,54,72,90]
Differential equation model	[24,56]
Digital elevation raster model (DEM)	[78]
Ecological niche factor analysis (ENFA)	[18,26,39,96]
Environmental niche model (ENM)	[93,106]
Epidemiological model	[113]
Generalized linear model (GLM)	[19,22,25,27,31,34,57,61,62,68,69,78,79,80,100,104,111]
Leslie models	[114,115,116]
Markov model	[110]
Maximum entropy (MaxEnt)	[20,28,37,44,45,47,50,58,65,92]
Multi-criteria decision analysis (MCDA)	[74]
Multi-habitat model	[67]
Next generation matrix (NGM) model	[55,60]
Process-driven model	[52]
Reaction-diffusion model	[29]
Species distribution modeling (SDM)	[42,51,78,97,98]
Weather research and forecasting (WRF) model	[36,89]
Weights-of-evidence method (WofE)	[26]

**Table 6:** Modeling *Rhipicephalus* (*Boophilus*) *microplus* in Africa between 2001 and 2020.

Year	Country	Models	References
2008	Tanzania	Ecological niche factor analysis (ENFA) Classification and regression tree (CART)	[96]
2009	Africa	Logistic regression	[100]
2013	Republic of Benin	Maximum entropy (MaxEnt)	[92]
2015	Republic of Benin	Generalized linear model (GLM) Linear discriminant analysis (LDA) Classification regression tree (CART)	[91]
2018	Zimbabwe	Generalized linear model (GLM)	[99]

**Table 7:** Inclusion and exclusion criteria.

Inclusion Criteria	Exclusion Criteria
Original articles written in English or French; Articles should be published in peer-reviewed journals during the last 20 years (from 2001 to 2020); Articles outlining quantitative and/or qualitative risk modeling of the spread or distribution of ticks and/or TBD; Studies on spatial and temporal distribution of ticks and TBD.	Studies focused on vectors and vector-borne diseases different from ticks and associated diseases; Studies related to ticks and TBD concerning only humans; Articles that describe biological models instead of statistical or mathematical ones; Articles focused on efficacy or resistance analysis of acaricides on ticks; Articles that did not address ticks and/or TBD distribution, and risk factors for their spread; Review articles on modeling the risk of spread or distribution of ticks and/or TBD; Studies related only to economic models; Studies related to vaccine efficacy models; Studies related to simulation models; Studies related to TBD transmission modeling.

TBD, tick-borne diseases.

## Data Availability

The data that support the findings of this survey are available from the corresponding author upon reasonable request.

## Appendix A

PRISMA checklist.

**Section/Topic**	**#**	**Checklist Item**	**Reported on Page #**
**Title**			
Title	1	Identify the report as a systematic review, meta-analysis, or both.	1
**Abstract**			
Structured summary	2	Provide a structured summary including, as applicable: background; objectives; data sources; study eligibility criteria, participants, and interventions; study appraisal and synthesis methods; results; limitations; conclusions and implications of key findings; systematic review registration number.	1
**Introduction**			
Rationale	3	Describe the rationale for the review in the context of what is already known.	2
Objectives	4	Provide an explicit statement of questions being addressed with reference to Participants, Interventions, Comparisons, Outcomes, and Study design (PICOS).	4
**Methods**			
Protocol and registration	5	Indicate if a review protocol exists, if and where it can be accessed (e.g., Web address), and, if available, provide registration information including registration number.	4
Eligibility criteria	6	Specify study characteristics (e.g., PICOS, length of follow-up) and report characteristics (e.g., years considered, language, publication status) used as criteria for eligibility, giving rationale.	4–5
Information sources	7	Describe all information sources (e.g., databases with dates of coverage, contact with study authors to identify additional studies) in the search and date last searched.	4–5
Search	8	Present full electronic search strategy for at least one database, including any limits used, such that it could be repeated.	4–5
Study selection	9	State the process for selecting studies (i.e., screening, eligibility, included in systematic review, and, if applicable, included in the meta-analysis).	4–5
Data collection process	10	Describe the method of data extraction from reports (e.g., piloted forms, independently, in duplicate) and any processes for obtaining and confirming data from investigators.	4–5
Data items	11	List and define all variables for which data were sought (e.g., PICOS, funding sources) and any assumptions and simplifications made.	4–5
Risk of bias in individualstudies	12	Describe methods used for assessing the risk of bias of individual studies (including specification of whether this was done at the study or outcome level), and how this information is to be used in any data synthesis.	5
Summary measures	13	State the principal summary measures (e.g., risk ratio, the difference in means).	5
Synthesis of results	14	Describe the methods of handling data and combining results of studies, if done, including measures of consistency (e.g., I2) for each meta-analysis.	5
Risk of bias across studies	15	Specify any assessment of the risk of bias that may affect the cumulative evidence (e.g., publication bias, selective reporting within studies).	5
Additional analyses	16	Describe methods of additional analyses (e.g., sensitivity or subgroup analyses, meta-regression), if done, indicating which were pre-specified.	5
**Results**			
Study selection	17	Give numbers of studies screened, assessed for eligibility, and included in the review, with reasons for exclusions at each stage, ideally with a flow diagram.	5–9
Study characteristics	18	For each study, present characteristics for which data were extracted (e.g., study size, PICOS, follow-up period) and provide the citations.	5–9
Risk of bias within studies	19	Present data on the risk of bias of each study and, if available, any outcome level assessment (see item 12).	9
Results of individual studies	20	For all outcomes considered (benefits or harms), present, for each study: (a) simple summary data for each intervention group (b) effect estimates and confidence intervals, ideally with a forest plot.	9
Synthesis of results	21	Present results of each meta-analysis done, including confidence intervals and measures of consistency.	9
Risk of bias across studies	22	Present results of any assessment of the risk of bias across studies (see Item 15).	9
Additional analysis	23	Give results of additional analyses, if done (e.g., sensitivity or subgroup analyses, meta-regression (see Item 16)).	7–9
**Discussion**			
Summary of evidence	24	Summarize the main findings including the strength of evidence for each main outcome; consider their relevance to key groups (e.g., healthcare providers, users, and policymakers).	9–15
Limitations	25	Discuss limitations at study and outcome level (e.g., risk of bias), and review-level (e.g., incomplete retrieval of identified research, reporting bias).	9–15
Conclusions	26	Provide a general interpretation of the results in the context of other evidence and implications for future research.	15
**Funding**			
Funding	27	Describe sources of funding for the systematic review and other support (e.g., supply of data); the role of funders for the systematic review.	16

## Appendix B

Search strategies and results for PubMed and Scopus databases

**Last Date of Search**	**Database Consulted**	**Search Algorithms Applied**	**Results**
28 September 2018	Scopus	((modelling or modeling or models) and (animals and ticks) and (tick and borne and diseases) and (distribution or spread) and dynamic)	901
28 September 2018	Pubmed	((modelling or modeling or models) and (animals and ticks) and (tick and borne and diseases) and (distribution or spread) and dynamic)	445
28 September 2018	Scopus	modelling the distribution dynamic of animals ticks AND tick borne disease	306
28 September 2018	Pubmed	modelling the distribution dynamic of animals ticks AND tick borne disease	128
28 September 2018	Scopus	qualitative modelling of the distribution dynamic of animals ticks AND tick borne disease	57
28 September 2018	Pubmed	qualitative modelling of the distribution dynamic of animals ticks AND tick borne disease	29
28 September 2018	Scopus	quantitative modelling of the distribution dynamic of animals ticks AND tick borne disease	183
28 September 2018	Pubmed	quantitative modelling of the distribution dynamic of animals ticks AND tick borne disease	63
28 September 2018	Scopus	animals ticks and tick-borne disease spread risk modelling	543
28 September 2018	Pubmed	animals ticks and tick-borne disease spread risk modelling	217
15 January 2021	Scopus	((modelling or modeling or models) and (animals and ticks) and (tick and borne and diseases) and (distribution or spread) and dynamic)	64
15 January 2021	Pubmed	((modelling or modeling or models) and (animals and ticks) and (tick and borne and diseases) and (distribution or spread) and dynamic)	36
All results for Scopus			2054
All results for Pubmed			918
Overall results			2972

## Appendix C

Classification of the models, which area under the curve (AUC) are available, based on J.A. Swets rules.

**Models**	**References**	**Accuracy**
Bayesian hierarchical model	[102]	Useful
Classification and regression tree (CART)	[17,91]	Highly accurate
	[17,33,71,77]	Useful
Ecological niche factor analysis	[39]	Highly accurate
	[39]	Useful
Environmental niche model (ENM)	[75,93]	Highly accurate
Generalized linear model (GLM)	[17,38,53,91]	Highly accurate
	[17,33,59,63,71,83,99,108,109]	Useful
Linear discriminant analysis (LDA)	[91]	Highly accurate
	[17]	Useful
Maximum entropy (MaxEnt)	[17,20,21,43,44,46,50,84,88,94,95,103,107]	Highly accurate
	[17,30,33,35,40,44,49,50,58,64,70,71]	Useful
	[44]	Poorly accurate
Multi-criteria decision analysis (MCDA)	[12]	Useful
Specie distribution modeling (SDM)	[17,41]	Useful
	[17]	Poorly accurate
Survival model	[76]	Useful

## References

[1] Rajput Z.I., Hu S., Chen W., Arijo A.G., Xiao C. (2006). Importance of ticks and their chemical and immunological control in livestock. J. Zhejiang Univ. Sci. B.

[2] Kivaria F.M. (2006). Estimated direct economic costs associated with tick-borne diseases on cattle in Tanzania. Trop. Anim. Health Prod..

[3] Jongejan F. (1999). Integrated Control of Ticks and Tick-Borne Diseases. Parassitologia.

[4] Madder M., Thys E., Geysen D., Baudoux C., Horak I. (2007). Boophilus microplus ticks found in West Africa. Exp. Appl. Acarol..

[5] Madder M., Adehan S., De Deken R., Adehan R., Lokossou R. (2012). New foci of Rhipicephalus microplus in West Africa. Exp. Appl. Acarol..

[6] Robinson S.J., Neitzel D.F., Moen R.A., Craft M.E., Hamilton K.E., Johnson L.B., Mulla D.J., Munderloh U.G., Redig P.T., Smith K.E. (2015). Disease Risk in a Dynamic Environment: The Spread of Tick-Borne Pathogens in Minnesota, USA. EcoHealth.

[7] Garner M., Dubé C., AStevenson M., Sanson R., Estrada C., Griffin J. (2007). Evaluating alternative approaches to managing animal disease outbreaks—The role of modelling in policy formulation. Vet. Ital..

[8] Singer A., Salman M., Thulke H.-H. (2011). Reviewing model application to support animal health decision making. Prev. Vet. Med..

[9] Taylor N. (2003). Review of the use of models in informing disease control policy development and adjustment. DEFRA UK.

[10] Requena-García F., Cabrero-Sañudo F., Olmeda-García S., González J., Valcárcel F. (2017). Influence of environmental temperature and humidity on questing ticks in central Spain. Exp. Appl. Acarol..

[11] Estrada-Peña A. (2008). Climate, niche, ticks, and models: What they are and how we should interpret them. Parasitol Res..

[12] Vial L., Ducheyne E., Filatov S., Gerilovych A., McVey D.S., Sindryakova I. (2018). Spatial multi-criteria decision analysis for modelling suitable habitats of Ornithodoros soft ticks in the Western Palearctic region. Vet. Parasitol..

[13] Elith J., Leathwick J.R. (2009). Species Distribution Models: Ecological Explanation and Prediction Across Space and Time. Annu. Rev. Ecol. Evol. Syst..

[14] Jacob S.S., Sengupta P.P., Paramanandham K., Suresh K.P., Chamuah J.K., Rudramurthy G.R., Roy P. (2020). Bovine babesiosis: An insight into the global perspective on the disease distribution by systematic review and meta-analysis. Vet. Parasitol..

[15] Rashid M., Rashid M.I., Akbar H., Ahmad L., Hassan M.A., Ashraf K., Saeed K., Gharbi M. (2019). A systematic review on modelling approaches for economic losses studies caused by parasites and their associated diseases in cattle. Parasitology.

[16] Bermúdez S.E., Castro A.M., Trejos D., García G.G., Gabster A., Miranda R.J., Zaldívar Y., Paternina L.E. (2016). Distribution of Spotted Fever Group Rickettsiae in Hard Ticks (Ixodida: Ixodidae) from Panamanian Urban and Rural Environments (2007–2013). EcoHealth.

[17] Clarke-Crespo E., Moreno-Arzate C.N., López-González C.A. (2020). Ecological Niche Models of Four Hard Tick Genera (Ixodidae) in Mexico. Animals.

[18] Estrada-Peña A. (2003). Climate change decreases habitat suitability for some tick species (Acari: Ixodidae) in South Africa. Onderstepoort J. Vet. Res..

[19] Estrada-Peña A., de la Fuente J., Cabezas-Cruz A. (2016). A comparison of the performance of regression models of *Amblyomma americanum* (L.) (Ixodidae) using life cycle or landscape data from administrative divisions. Ticks Tick-Borne Dis..

[20] Estrada-Peña A., Tarragona E.L., Vesco U., Meneghi D.D., Mastropaolo M., Mangold A.J., Guglielmone A.A., Nava S. (2014). Divergent environmental preferences and areas of sympatry of tick species in the Amblyomma cajennense complex (Ixodidae). Int. J. Parasitol..

[21] Estrada-Peña A., Horak I.G., Petney T. (2008). Climate changes and suitability for the ticks Amblyomma hebraeum and Amblyomma variegatum (Ixodidae) in Zimbabwe (1974–1999). Vet. Parasitol..

[22] Ferrell A.M., Brinkerhoff R.J., Bernal J., Bermúdez S.E. (2017). Ticks and tick-borne pathogens of dogs along an elevational and land-use gradient in Chiriquí province, Panamá. Exp. Appl. Acarol..

[23] Gaff H.D. (2011). Preliminary analysis of an agent-based model for a tick-borne disease. Math. Biosci. Eng..

[24] Gaff H.D., Gross L.J. (2007). Modeling Tick-Borne Disease: A Metapopulation Model. Bull. Math. Biol..

[25] Kaizer A.M., Foré S.A., Kim H.-J., York E.C. (2015). Modeling the biotic and abiotic factors that describe the number of active off-host Amblyomma americanum larvae. J Vector Ecol..

[26] Lynen G., Zeman P., Bakuname C., Di Giulio G., Mtui P., Sanka P., Jongejan F. (2007). Cattle ticks of the genera Rhipicephalus and Amblyomma of economic importance in Tanzania: Distribution assessed with GIS based on an extensive field survey. Exp. Appl. Acarol..

[27] Miguel E., Boulinier T., de Garine-Wichatitsky M., Caron A., Fritz H., Grosbois V. (2014). Characterising African tick communities at a wild-domestic interface using repeated sampling protocols and models. Acta Trop..

[28] Oliveira S.V.D., Romero-Alvarez D., Martins T.F., Santos J.P.D., Labruna M.B., Gazeta G.S., Escobar L.E., Gurgel-Gonçalves R. (2017). Amblyomma ticks and future climate: Range contraction due to climate warming. Acta Trop..

[29] Polo G., Acosta C.M., Labruna M.B., Ferreira F., Brockmann D. (2018). Hosts mobility and spatial spread of *Rickettsia rickettsii*. PLoS Comput. Biol..

[30] Raghavan R.K., Goodin D.G., Hanzlicek G.A., Zolnerowich G., Dryden M.W., Anderson G.A., Ganta R.R. (2016). Maximum entropy-based ecological niche model and bio-climatic determinants of lone star tick (*Amblyomma americanum*) niche. Vector Borne Zoonotic Dis..

[31] Sagurova I., Ludwig A., Ogden N.H., Pelcat Y., Dueymes G., Gachon P. (2019). Predicted Northward Expansion of the Geographic Range of the Tick Vector Amblyomma americanum in North America under Future Climate Conditions. Environ. Health Perspect..

[32] Simpson D.T., Teague M.S., Weeks J.K., Kaup B.Z., Kerscher O., Leu M. (2019). Habitat amount, quality, and fragmentation associated with prevalence of the tick-borne pathogen Ehrlichia chaffeensis and occupancy dynamics of its vector, Amblyomma americanum. Landsc. Ecol..

[33] Springer Y.P., Jarnevich C.S., Barnett D.T., Monaghan A.J., Eisen R.J. (2015). Modeling the present and future geographic distribution of the lone star tick, amblyomma americanum (ixodida: Ixodidae), in the continental United States. Am. J. Trop. Med. Hyg..

[34] Stein K.J., Waterman M., Waldon J.L. (2008). The effects of vegetation density and habitat disturbance on the spatial distribution of ixodid ticks (acari: Ixodidae). Geospat. Health.

[35] Atkinson S.F., Sarkar S., Aviña A., Schuermann J.A., Williamson P. (2013). Modelling spatial concordance between Rocky Mountain spotted fever disease incidence and habitat probability of its vector Dermacentor variabilis (American dog tick). Geospat. Health.

[36] Beugnet F., Chalvet-Monfray K., Loukos H. (2009). FleaTickRisk: A meteorological model developed to monitor and predict the activity and density of three tick species and the cat flea in Europe. Geospat. Health.

[37] Boorgula G.D.Y., Peterson A.T., Foley D.H., Ganta R.R., Raghavan R.K. (2020). Assessing the current and future potential geographic distribution of the American dog tick, Dermacentor variabilis (Say) (Acari: Ixodidae) in North America. PLoS ONE.

[38] Eisen L., Eisen R.J., Lane R.S. (2006). Geographical distribution patterns and habitat suitability models for presence of host-seeking ixodid ticks in dense woodlands of Mendocino County, California. J. Med. Entomol..

[39] Estrada-Peña A., Venzal J.M. (2007). Climate niches of tick species in the mediterranean region: Modeling of occurrence data, distributional constraints, and impact of climate change. J. Med. Entomol..

[40] Huercha, Song R., Ma Y., Hu Z., Li Y., Li M., Wu L., Li C., Dao E., Fan X. (2020). MaxEnt Modeling of Dermacentor marginatus (Acari: Ixodidae) Distribution in Xinjiang, China. J. Med. Entomol..

[41] Minigan J.N., Hager H.A., Peregrine A.S., Newman J.A. (2018). Current and potential future distribution of the American dog tick (Dermacentor variabilis, Say) in North America. Ticks Tick-Borne Dis..

[42] Široký P., Kubelová M., Bednář M., Modrý D., Hubálek Z., Tkadlec E. (2011). The distribution and spreading pattern of Dermacentor reticulatus over its threshold area in the Czech Republic-How much is range of this vector expanding?. Vet. Parasitol..

[43] John H.K., Adams M.L., Masuoka P.M., Flyer-Adams J.G., Jiang J., Rozmajzl P.J., Stromdahl E.Y., Richards A.L. (2016). Prevalence, Distribution, and Development of an Ecological Niche Model of Dermacentor variabilis Ticks Positive for Rickettsia montanensis. Vector Borne Zoonotic Dis..

[44] Williams H.W., Cross D.E., Crump H.L., Drost C.J., Thomas C.J. (2015). Climate suitability for European ticks: Assessing species distribution models against null models and projection under AR5 climate. Parasites Vectors.

[45] Lawrence K.E., Summers S.R., Heath A.C.G., McFadden A.M.J., Pulford D.J., Tait A.B., Pomroy W.E. (2017). Using a rule-based envelope model to predict the expansion of habitat suitability within New Zealand for the tick Haemaphysalis longicornis, with future projections based on two climate change scenarios. Vet. Parasitol..

[46] Lawrence K.E., Summers S.R., Heath A.C.G., McFadden A.M.J., Pulford D.J., Pomroy W.E. (2016). Predicting the potential environmental suitability for Theileria orientalis transmission in New Zealand cattle using maximum entropy niche modelling. Vet. Parasitol..

[47] Raghavan R.K., Barker S.C., Cobos M.E., Barker D., Teo E.J.M., Foley D.H., Nakao R., Lawrence K., Heath A.C.G., Pe-terson A.T. (2019). Potential Spatial Distribution of the Newly Introduced Long-horned Tick, Haemaphysalis longicornis in North America. Sci. Rep..

[48] Bosch J., Muñoz M.J., Martínez M., de la Torre A., Estrada-Peña A. (2013). Vector-Borne pathogen spread through ticks on migratory birds: A probabilistic spatial risk model for south-western europe. Transboundary Emer Dis..

[49] Deka M.A. (2018). Crimean-Congo Hemorrhagic Fever Geographic and Environmental Risk Assessment in the Balkan and Anatolian Peninsulas. Pap. Appl. Geogr..

[50] Domşa C., Sándor A.D., Mihalca A.D. (2016). Climate change and species distribution: Possible scenarios for thermophilic ticks in Romania. Geospat. Health.

[51] Estrada-Peña A., Alexander N., Wint G.R.W. (2016). Perspectives on modelling the distribution of ticks for large areas: So far so good?. Parasites Vectors.

[52] Estrada-Peña A., Sánchez N., Estrada-Sánchez A. (2012). An assessment of the distribution and spread of the tick hyalomma marginatum in the western palearctic under different climate scenarios. Vector Borne Zoonotic Dis..

[53] Brownstein J.S., Holford T.R., Fish D. (2003). A climate-based model predicts the spatial distribution of the Lyme disease vector Ixodes scapularis in the United States. Environ. Health Perspect..

[54] Ogden N.H. (2005). A dynamic population model to investigate effects of climate on geographic range and seasonality of the tick Ixodes scapularis. Int. J. Parasitol..

[55] Dunn J.M., Davis S., Stacey A., Diuk-Wasser M.A. (2013). A simple model for the establishment of tick-borne pathogens of Ixodes scapularis: A global sensitivity analysis of R0. J. Ther. Biol..

[56] Cheng A., Chen D., Woodstock K., Ogden N.H., Wu X., Wu J. (2017). Analyzing the potential risk of climate change on lyme disease in Eastern Ontario, Canada using time series remotely sensed temperature data and tick population modelling. Remote Sens..

[57] Bolzoni L., Rosà R., Cagnacci F., Rizzoli A. (2012). Effect of deer density on tick infestation of rodents and the hazard of tick-borne encephalitis. II: Population and infection models. Int. J. Parasitol..

[58] Porretta D., Mastrantonio V., Amendolia S., Gaiarsa S., Epis S., Genchi C., Bandi C., Otranto D., Urbanelli S. (2013). Effects of global changes on the climatic niche of the tick Ixodes ricinus inferred by species distribution modelling. Parasites Vectors.

[59] Estrada-Peña A. (2005). Effects of habitat suitability and landscape patterns on tick (Acarina) metapopulation processes. Landsc. Ecol..

[60] Rosà R., Pugliese A. (2007). Effects of tick population dynamics and host densities on the persistence of tick-borne infections. Math Biosci..

[61] Boehnke D., Brugger K., Pfäffle M., Sebastian P., Norra S., Petney T., Oehme R., Littwin N., Lebl K., Raith J. (2015). Estimating Ixodes ricinus densities on the landscape scale. Int. J. Health Geogr..

[62] Ruiz-Fons F., Fernández-de-Mera I.G., Acevedo P., Gortázar C., de la Fuente J. (2012). Factors driving the abundance of Ixodes ricinus ticks and the prevalence of zoonotic I. ricinus-borne pathogens in natural foci. Appl. Environ. Microbiol..

[63] Diuk-Wasser M.A., Vourc’h G., Cislo P., Hoen A.G., Melton F., Hamer S.A., Rowland M., Cortinas R., Hickling G.J., Tsao J.I. (2010). Field and climate-based model for predicting the density of host-seeking nymphal Ixodes scapularis, an important vector of tick-borne disease agents in the eastern United States. Glob. Ecol. Biogeogr..

[64] Johnson T.L., Bjork J.K.H., Neitzel D.F., Dorr F.M., Schiffman E.K., Eisen R.J. (2016). Habitat suitability model for the distribution of Ixodes scapularis (acari: Ixodidae) in Minnesota. J. Med. Entomol..

[65] Estrada-Peña A., de la Fuente J. (2017). Host distribution does not limit the range of the tick ixodes ricinus but impacts the circulation of transmitted pathogens. Front. Cell Infect. Microbiol..

[66] Li S., Vanwambeke S.O., Licoppe A.M., Speybroeck N. (2014). Impacts of deer management practices on the spatial dynamics of the tick Ixodes ricinus: A scenario analysis. Ecol. Model..

[67] Hoch T., Monnet Y., Agoulon A. (2010). Influence of host migration between woodland and pasture on the population dynamics of the tick Ixodes ricinus: A modelling approach. Ecol. Model..

[68] Cat J., Beugnet F., Hoch T., Jongejan F., Prangé A., Chalvet-Monfray K. (2017). Influence of the spatial heterogeneity in tick abundance in the modeling of the seasonal activity of Ixodes ricinus nymphs in Western Europe. Exp. Appl. Acarol..

[69] Ogden N.H., Trudel L., Artsob H., Barker I.K., Beauchamp G., Charron D.F., Drebot M.A., Galloway T.D., O’handley R., Thompson R.A. (2006). Ixodes scapularis Ticks Collected by Passive Surveillance in Canada: Analysis of Geographic Distribution and Infection with Lyme Borreliosis Agent Borrelia burgdorferi. J. Med. Entomol..

[70] Estrada-Peña A., Estrada-Sánchez A., Estrada-Sánchez D. (2015). Methodological caveats in the environmental modelling and projections of climate niche for ticks, with examples for Ixodes ricinus (Ixodidae). Vet. Parasitol..

[71] Hahn M.B., Jarnevich C.S., Monaghan A.J., Eisen R.J. (2016). Modeling the Geographic Distribution of Ixodes scapularis and Ixodes pacificus (Acari: Ixodidae) in the Contiguous United States. J. Med. Entomol..

[72] Hoch T., Goebel J., Agoulon A., Malandrin L. (2012). Modelling bovine babesiosis: A tool to simulate scenarios for pathogen spread and to test control measures for the disease. Prev. Vet. Med..

[73] Li S., Gilbert L., Harrison P.A., Rounsevell M.D.A. (2016). Modelling the seasonality of Lyme disease risk and the potential impacts of a warming climate within the heterogeneous landscapes of Scotland. J. R. Soc. Interface.

[74] Rousseau R., McGrath G., McMahon B.J., Vanwambeke S.O. (2017). Multi-criteria Decision Analysis to Model Ixodes ricinus Habitat Suitability. EcoHealth.

[75] Boeckmann M., Joyner T.A. (2014). Old health risks in new places? An ecological niche model for I. ricinus tick distribution in Europe under a changing climate. Health Place.

[76] Leighton P.A., Koffi J.K., Pelcat Y., Lindsay L.R., Ogden N.H. (2012). Predicting the speed of tick invasion: An empirical model of range expansion for the Lyme disease vector Ixodes scapularis in Canada. J. Appl. Ecol..

[77] Alfredsson M., Olafsson E., Eydal M., Unnsteinsdottir E.R., Hansford K., Wint W., Alexander N., Medlock J.M. (2017). Surveillance of Ixodes ricinus ticks (Acari: Ixodidae) in Iceland. Parasites Vectors.

[78] Qviller L., Viljugrein H., Loe L.E., Meisingset E.L., Mysterud A. (2016). The influence of red deer space use on the distribution of Ixodes ricinus ticks in the landscape. Parasites Vectors.

[79] Tomkins J.L., Aungier J., Hazel W., Gilbert L. (2014). Towards an evolutionary understanding of questing behaviour in the tick Ixodes ricinus. PLoS ONE.

[80] Barrios J.M., Verstraeten W.W., Maes P., Aerts J.-M., Farifteh J., Coppin P. (2012). Using the gravity model to estimate the spatial spread of vector-borne diseases. Int. J. Environ. Res. Public Health.

[81] Ostfeld R.S., Canham C.D., Oggenfuss K., Winchcombe R.J., Keesing F. (2006). Climate, Deer, Rodents, and Acorns as Determinants of Variation in Lyme-Disease Risk. Dobson A, editor. PLoS Biol..

[82] Wu X., Duvvuri V.R., Lou Y., Ogden N.H., Pelcat Y., Wu J. (2013). Developing a temperature-driven map of the basic reproductive number of the emerging tick vector of Lyme disease Ixodes scapularis in Canada. J. Theor. Biol..

[83] Lieske D.J., Lloyd V.K. (2018). Combining public participatory surveillance and occupancy modelling to predict the distributional response of Ixodes scapularis to climate change. Ticks Tick-Borne Dis..

[84] Zanet S., Ferroglio E., Battisti E., Tizzani P. (2020). Ecological niche modeling of Babesia sp infection in wildlife experimentally evaluated in questing Ixodes ricinus. Geospat. Health.

[85] Li S., Gilbert L., Vanwambeke S.O., Yu J., Purse B.V., Harrison P.A. (2019). Lyme Disease Risks in Europe under Multiple Uncertain Drivers of Change. Environ. Health Perspect..

[86] Nguyen A., Mahaffy J., Vaidya N.K. (2019). Modeling transmission dynamics of lyme disease: Multiple vectors, seasonality, and vector mobility. Infect. Dis. Model..

[87] Kjær L.J., Soleng A., Edgar K.S., Lindstedt H.E.H., Paulsen K.M., Andreassen Å.K., Korslund L., Kjelland V., Slettan A., Stuen S. (2019). Predicting and mapping human risk of exposure to Ixodes ricinus nymphs using climatic and environmental data, Denmark, Norway and Sweden, 2016. Euro Surveill..

[88] Slatculescu A.M., Clow K.M., McKay R., Talbot B., Logan J.J., Thickstun C.R., Jardine C.M., Ogden N.H., Knudby A.J., Kulkarni M.A. (2020). Species distribution models for the eastern blacklegged tick, Ixodes scapularis, and the Lyme disease pathogen, Borrelia burgdorferi, in Ontario, Canada. PLoS ONE.

[89] Beugnet F., Kolasinski M., Michelangeli P.-A., Vienne J., Loukos H. (2011). Mathematical modelling of the impact of climatic conditions in France on Rhipicephalus sanguineus tick activity and density since 1960. Geospat. Health.

[90] Corson M.S., Teel P.D., Grant W.E. (2004). Microclimate influence in a physiological model of cattle-fever tick (Boophilus spp.) population dynamics. Ecol. Model..

[91] De Clercq E.M., Leta S., Estrada-Peña A., Madder M., Adehan S., Vanwambeke S.O. (2015). Species distribution modelling for Rhipicephalus microplus (Acari: Ixodidae) in Benin, West Africa: Comparing datasets and modelling algorithms. Prev. Vet. Med..

[92] De Clercq E.M., Estrada-Peña A., Adehan S., Madder M., Vanwambeke S.O. (2013). An update on distribution models for Rhipicephalus microplus in West Africa. Geospat. Health.

[93] Estrada-Peña A., Sánchez Acedo C., Quílez J., Del Cacho E. (2005). A retrospective study of climatic suitability for the tick Rhipicephalus (Boophilus) microplus in the Americas. Glob. Ecol. Biogeogr..

[94] Hadgu M., Menghistu H.T., Girma A., Abrha H., Hagos H. (2019). Modeling the potential climate change- induced impacts on future genus Rhipicephalus (Acari: Ixodidae) tick distribution in semi-arid areas of Raya Azebo district, Northern Ethiopia. J. Ecol. Environ..

[95] Leta S., De Clercq E.M., Madder M. (2013). High-resolution predictive mapping for Rhipicephalus appendiculatus (Acari: Ixodidae) in the Horn of Africa. Exp. Appl. Acarol..

[96] Lynen G., Zeman P., Bakuname C., Di Giulio G., Mtui P., Sanka P., Jongejan F. (2008). Shifts in the distributional ranges of Boophilus ticks in Tanzania: Evidence that a parapatric boundary between Boophilus microplus and B. decoloratus follows climate gradients. Exp. Appl. Acarol..

[97] Olwoch J.M., Reyers B., Engelbrecht F.A., Erasmus B.F.N. (2008). Climate change and the tick-borne disease, Theileriosis (East Coast fever) in sub-Saharan Africa. J. Arid Environ..

[98] Olwoch J.M., Van Jaarsveld A.S., Scholtz C.H., Horak I.G. (2007). Climate change and the genus rhipicephalus (Acari: Ixodidae) in Africa. Onderstepoort J. Vet. Res..

[99] Sungirai M., Moyo D.Z., De Clercq P., Madder M., Vanwambeke S.O., De Clercq E.M. (2018). Modelling the distribution of Rhipicephalus microplus and R. decoloratus in Zimbabwe. Veterinary Parasitology. Reg. Stud. Rep..

[100] Sutherst R.W., Bourne A.S. (2009). Modelling non-equilibrium distributions of invasive species: A tale of two modelling paradigms. Biol. Invasions.

[101] Liu Y., Watson S.C., Gettings J.R., Lund R.B., Nordone S.K., Yabsley M.J., McMahan C.S. (2017). A Bayesian spatio-temporal model for forecasting Anaplasma species seroprevalence in domestic dogs within the contiguous United States. PLoS ONE.

[102] Wimberly M.C., Baer A.D., Yabsley M.J. (2008). Enhanced spatial models for predicting the geographic distributions of tick-borne pathogens. Int. J. Health Geogr..

[103] Kouam M.K., Masuoka P.M., Kantzoura V., Theodoropoulos G. (2010). Geographic distribution modeling and spatial cluster analysis for equine piroplasms in Greece. Infec. Genet. Evol..

[104] Delgado J.D., Abreu-Yanes E., Abreu-Acosta N., Flor M.D., Foronda P. (2017). Vertebrate ticks distribution and their role as vectors in relation to road edges and underpasses. Vector Borne Zoonotic Dis..

[105] Watson S.C., Liu Y., Lund R.B., Gettings J.R., Nordone S.K., McMahan C.S., Yabsley M.J. (2017). A Bayesian spatio-temporal model for forecasting the prevalence of antibodies to Borrelia burgdorferi, causative agent of Lyme disease, in domestic dogs within the contiguous United States. PLoS ONE.

[106] Machado G., Korennoy F., Alvarez J., Picasso-Risso C., Perez A., VanderWaal K. (2019). Mapping changes in the spatiotemporal distribution of lumpy skin disease virus. Transbound. Emerg. Dis..

[107] Nogareda C., Jubert A., Kantzoura V., Kouam M.K., Feidas H., Theodoropoulos G. (2013). Geographical distribution modelling for Neospora caninum and Coxiella burnetii infections in dairy cattle farms in northeastern Spain. Epidemiol. Infect..

[108] Raghavan R.K., Almes K., Goodin D.G., Harrington J.A., Stackhouse P.W. (2014). Spatially heterogeneous land cover/land use and climatic risk factors of tick-borne feline cytauxzoonosis. Vector Borne Zoonotic Dis..

[109] Wimberly M.C., Yabsley M.J., Baer A.D., Dugan V.G., Davidson W.R. (2008). Spatial heterogeneity of climate and land-cover constraints on distributions of tick-borne pathogens. Glob. Ecol. Biogeogr..

[110] Nah K., Magpantay F.M.G., Bede-Fazekas Á., Röst G., Trájer A.J., Wu X., Zhang X., Wu J. (2019). Assessing systemic and non-systemic transmission risk of tick-borne encephalitis virus in Hungary. PLoS ONE.

[111] Mostafavi E., Chinikar S., Bokaei S., Haghdoost A. (2013). Temporal modeling of Crimean-Congo hemorrhagic fever in eastern Iran. Int. J. Infect. Dis..

[112] Liu Y., Lund R.B., Nordone S.K., Yabsley M.J., McMahan C.S. (2017). A Bayesian spatio-temporal model for forecasting the prevalence of antibodies to Ehrlichia species in domestic dogs within the contiguous United States. Parasites Vectors.

[113] Gilioli G., Groppi M., Vesperoni M.P., Baumgärtner J., Gutierrez A.P. (2009). An epidemiological model of East Coast Fever in African livestock. Ecol. Model..

[114] Liu K., Lou Y., Wu J. (2017). Analysis of an age structured model for tick populations subject to seasonal effects. J. Differ. Equ..

[115] Dobson A.D.M., Auld S.K.J.R. (2016). Epidemiological implications of host biodiversity and vector biology: Key insights from simple models. Am. Nat..

[116] Cobbold C.A., Teng J., Muldowney J.S. (2015). The influence of host competition and predation on tick densities and management implications. Theor. Ecol..

[117] Guisan A., Edwards T.C., Hastie T. (2002). Generalized linear and generalized additive models in studies of species distributions: Setting the scene. Ecol. Model..

[118] Phillips S.J., Dudík M. (2008). Modeling of species distributions with Maxent: New extensions and a comprehensive evaluation. Ecography.

[119] Phillips S.J., Anderson R.P., Schapire R.E. (2006). Maximum entropy modeling of species geographic distributions. Ecol. Model..

[120] Phillips S.J., Dudík M., Schapire R.E. (2004). A Maximum Entropy Approach to Species Distribution Modeling.

[121] Baldwin R.A. (2009). Use of maximum entropy modeling in wildlife research. Entropy.

[122] Baldwin R.A., Bender L.C. (2008). Den-Site Characteristics of Black Bears in Rocky Mountain National Park, Colorado. J. Wildl. Manag..

[123] Hoenes B.D., Bender L.C. (2010). Relative habitat-and browse-use of native desert mule deer and exotic oryx in the greater San Andres Mountains, New Mexico. Hum. Wildl. Interact..

[124] Yost A.C., Petersen S.L., Gregg M., Miller R. (2008). Predictive modeling and mapping sage grouse (Centrocercus urophasianus) nesting habitat using Maximum Entropy and a long-term dataset from Southern Oregon. Ecol. Inform..

[125] Cheng Z., Nakatsugawa M., Hu C., Robertson S.P., Hui X., Moore J.A., Bowers M.R., Kiess A.P., Page B.R., Burns L. (2018). Evaluation of classification and regression tree (CART) model in weight loss prediction following head and neck cancer radiation therapy. Adv. Radiat. Oncol..

[126] Batista G.E.A.P.A., Monard M.C. (2003). An analysis of four missing data treatment methods for supervised learning. Appl. Artif. Intell..

[127] Norman R., Bowers R.G., Begon M., Hudson P.J. (1999). Persistence of Tick-borne Virus in the Presence of Multiple Host Species: Tick Reservoirs and Parasite Mediated Competition. J. Theor. Biol..

[128] Pugliese A., Rosà R. (2008). Effect of host populations on the intensity of ticks and the prevalence of tick-borne pathogens: How to interpret the results of deer exclosure experiments. Parasitology.

[129] Maliyoni M., Chirove F., Gaff H.D., Govinder K.S. (2017). A Stochastic Tick-Borne Disease Model: Exploring the Probability of Pathogen Persistence. Bull. Math. Biol..

[130] Hussain S., Hussain A., Ho J., Li J., George D., Rehman A., Zeb J., Sparagano O. (2021). An Epidemiological Survey Regarding Ticks and Tick-Borne Diseases among Livestock Owners in Punjab, Pakistan: A One Health Context. Pathogens.

[131] Kardjadj M., Diallo A., Lancelot R. (2019). Transboundary Animal Diseases in Sahelian Africa and Connected Regions.

[132] Silatsa B.A., Simo G., Githaka N., Mwaura S., Kamga R.M., Oumarou F., Keambou C., Bishop R.P., Djikeng A., Kuiate J.-R. (2019). A comprehensive survey of the prevalence and spatial distribution of ticks infesting cattle in different agro-ecological zones of Cameroon. Parasites Vectors.

[133] Gustafson R., Jaenson T.G.T., Gardulf A., Mejlon H., Svenungsson B. (1995). Prevalence of Borrelia burgdorferi sensu lato Infection in Ixodes ricinus in Sweden. Scand. J. Infect. Dis..

[134] TaLleklint L., Jaenson T.G.T. (1996). Relationship Between Ixodes ricinus Density and Prevalence of Infection with Borrelia-Like Spirochetes and Density of Infected Ticks. J. Med. Entomol..

[135] Mejlon H.A., Jaenson T.G. (1993). Seasonal prevalence of Borrelia burgdorferi in Ixodes ricinus in different vegetation types in Sweden. Scand. J. Infect. Dis..

[136] Randolph S.E., Rogers D.J. (1997). A generic population model for the African tick Rhipicephalus appendiculatus. Parasitology.

[137] Perry B.D., Kruska R., Lessard P., Norval R.A.I., Kundert K. (1991). Estimating the distribution and abundance of *Rhipicephalus appendiculatus* in Africa. Prev. Vet. Med..

[138] Adakal H., Biguezoton A., Zoungrana S., Courtin F., De Clercq E.M., Madder M. (2013). Alarming spread of the Asian cattle tick Rhipicephalus microplus in West Africa—another three countries are affected: Burkina Faso, Mali and Togo. Exp. Appl. Acarol..

[139] Tuppurainen E.S.M., Oura C.A.L. (2012). Review: Lumpy Skin Disease: An Emerging Threat to Europe, the Middle East and Asia: Emerging Lumpy Skin Disease. Transbound. Emerg. Dis..

[140] Walker A., Bouattour A., Camicas J.-L. (2003). Ticks of Domestic Animals in Africa: A Guide to Identification of Species.

[141] Deem S.L., Perry B.D., Katende J.M., McDermott J.J., Mahan S.M., Maloo S.H., Morzaria S.P., Musoke A.J., Rowlands G.J. (1993). Variations in prevalence rates of tick-borne diseases in Zebu cattle by agroecological zone: Implications for East Coast fever immunization. Prev. Vet. Med..

[142] Perry B.D. (2009). Economic impacts of tick-borne diseases in Africa. Onderstepoort J. Vet. Res..

[143] Fielding A.H., Bell J.F. (1997). A review of methods for the assessment of prediction errors in conservation presence/absence models. Environ. Conserv..

[144] Swets J. (1988). Measuring the accuracy of diagnostic systems. Science.

[145] Guisan A., Graham C.H., Elith J., Huettmann F. (2007). The NCEAS Species Distribution Modelling Group. Sensitivity of predictive species distribution models to change in grain size. Divers. Distrib..

[146] Deeks J.J. (2001). Systematic reviews of evaluations of diagnostic and screening tests. BMJ.

[147] Higgins J.P.T., Thompson S.G. (2004). Controlling the risk of spurious findings from meta-regression. Stat. Med..

[148] Higgins J.P.T. (2003). Measuring inconsistency in meta-analyses. BMJ.

[149] Field A.P., Gillett R. (2010). How to do a meta-analysis. Br. J. Math. Stat. Psychol..

[150] Borenstein M., Higgins J.P.T. (2013). Meta-Analysis and Subgroups. Prev. Sci..

